# Characterising the latent structure and organisation of self-reported thoughts, feelings and behaviours in adolescents and young adults

**DOI:** 10.1371/journal.pone.0175381

**Published:** 2017-04-12

**Authors:** Michelle C. St Clair, Sharon Neufeld, Peter B. Jones, Peter Fonagy, Edward T. Bullmore, Raymond J. Dolan, Michael Moutoussis, Umar Toseeb, Ian M. Goodyer

**Affiliations:** 1 Department of Psychology, University of Bath, Bath, United Kingdom; 2 Department of Psychiatry, University of Cambridge, Cambridge, United Kingdom; 3 Division of Psychology and Language Sciences, University College London, London, United Kingdom; 4 Max Planck UCL Centre for Computational Psychiatry and Ageing, University College London, London, United Kingdom; 5 Wellcome Trust Centre for Neuroimaging, University College London, London, United Kingdom; 6 Department of Psychology, Manchester Metropolitan University, Manchester, United Kingdom; Radboud Universiteit, NETHERLANDS

## Abstract

Little is known about the underlying relationships between self-reported mental health items measuring both positive and negative emotional and behavioural symptoms at the population level in young people. Improved measurement of the full range of mental well-being and mental illness may aid in understanding the aetiological substrates underlying the development of both mental wellness as well as specific psychiatric diagnoses. A general population sample aged 14 to 24 years completed self-report questionnaires on anxiety, depression, psychotic-like symptoms, obsessionality and well-being. Exploratory and confirmatory factor models for categorical data and latent profile analyses were used to evaluate the structure of both mental wellness and illness items. First order, second order and bifactor structures were evaluated on 118 self-reported items obtained from 2228 participants. A bifactor solution was the best fitting latent variable model with one general latent factor termed ‘distress’ and five ‘distress independent’ specific factors defined as self-confidence, antisocial behaviour, worry, aberrant thinking, and mood. Next, six distinct subgroups were derived from a person-centred latent profile analysis of the factor scores. Finally, concurrent validity was assessed using information on hazardous behaviours (alcohol use, substance misuse, self-harm) and treatment for mental ill health: both discriminated between the latent traits and latent profile subgroups. The findings suggest a complex, multidimensional mental health structure in the youth population rather than the previously assumed first or second order factor structure. Additionally, the analysis revealed a low hazardous behaviour/low mental illness risk subgroup not previously described. Population sub-groups show greater validity over single variable factors in revealing mental illness risks. In conclusion, our findings indicate that the structure of self reported mental health is multidimensional in nature and uniquely finds improved prediction to mental illness risk within person-centred subgroups derived from the multidimensional latent traits.

## Introduction

Very few studies have concurrently investigated the underlying structure of mental ill health and mental well-being in young people. The psychopathology literature on mental illness symptom structure has, on the whole, remained distinct from the wider concepts of the structure of mental well-being. Indeed, the more common approach is to characterise mental illness alone through well-established symptom combinations [1], which rely on the assumption that the absence of symptoms is equivalent to no mental illness. However, the absence of mental illness symptoms cannot be taken as either necessary or sufficient evidence of positive mental health. Mental health or well-being has been defined as a state of positive feelings and functioning, which can either be present or absent without necessarily having any implications on an individual’s mental illness status [2]. Failing to account for mental well-being when examining the structure of thoughts, feelings and behaviours in young people, is likely to provide a skewed view of mental health in the population at large [3].

Currently the natural relationships of item level thoughts, feelings and behaviours in the youth community are largely unknown. Adolescence and young adulthood is both a time of substantial maturational changes within physiological and neurological systems [4,5] as well as correspondingly large social changes, both of which are reflected in a rise in the incidence of mental illness diagnoses and mental distress symptoms. Thus, this is a key developmental time to investigate the emerging relationships between thoughts, feelings and behaviours that may discriminate well-being from emerging abnormal mental states. To the authors’ knowledge, no study has investigated the structure of a wide range of mental health difficulties alongside mental well-being items, which is the focus of the current study.

In those studies that have investigated the structure of both positive and negative mental states together the results are rather mixed: some find separate factors for positive mental well-being and mental illness while others report a single higher order mental state factor and lower level factors (e.g, [6–9]). One study found a single general factor from all items, with specific factors for positive and negative mental health [10]. Despite variations in item pool and method of item selection, these prior studies report substantial correlations between the positive and negative mental state factors suggesting a close relationship between the constructs of positive mental well-being and mental illness.

With regards to negative mental states, the higher order structure of psychiatric disorders as well as mental illness symptoms that explain item relationships and diagnostic comorbidity have been extensively reported in adults and younger populations (e.g., [11–15]). Within child and adolescent psychiatry much research has evaluated higher order externalising (i.e., Conduct Disorder, Oppositional Defiant Disorder) and internalising (i.e., Depression, Anxiety) domains (e.g, [13,16]) as a way of accounting for the substantial comorbidity between psychiatric diagnoses. Recent research has also indicated higher order factors relating to thought disorders and pathological introversion, composed of social anxiety, dependence and unassertive traits [14]. Much previous literature (in both mental wellness and illness) has assumed that first order factors (such as externalising and internalising) are orthogonal or uncorrelated factors of equal potential value. However, high correlations between these factors led to investigations of a single general mental illness factor that could be common to all indicators in addition to the lower level domains [17,18]. There is a growing research base that has re-examined the structure of symptoms using diagnoses and/or item level data and identified a single common “psychopathology” factor [17–26]. Initial work has been conducted which combined mental well-being and illness items within a similar analytic framework and also found evidence for a single overarching factor [limitations as described above; 10,see also [27] where prosocial items were included alongside mental illness items, in a younger population (7–14 years of age)]. Therefore, there is evidence to suggest that utilising a larger range of items encompassing mental health (well-being and illness) may generate a general distress or “psychopathology” factor together with more detailed specific factors implying a more multi-dimensional nature to thoughts feelings and behaviours in the youth population than considered hitherto.

The pathogenesis viewpoint on measuring mental illness has, understandably, focused on case-control studies of the mentally ill as compared to the not mentally ill, with the latter defined by the absence of predefined illness criteria rather than by the presence of a positive mental state or well-being. Recently, however, there has been a shift to focus research away from clinical types and towards quantitative measures of behaviour, cognition, physiology, and genetic factors [28,29]. This is driven in part by the low validity inherent in the descriptive diagnostic system and the need to develop a greater understanding of the aetiologies and pathophysiologies of mental illnesses [30]. Redefining how we conceptualise the ‘surface’ characteristics of mental health in the population at large using both mental illness and mental wellness items may create a more accurate behavioural phenotype of mental states in the natural world [31]. This may in turn translate to greater precision in pinpointing the relevant genetic, neural and cognitive factors that could underlie mentally well together with ill states. Just as psychopathology extends beyond diagnoses into the general population so, too, may the biological processes revealed by this approach, revealing a richer pattern of variance than exposed through a binary approach. A broader approach appears to be warranted: Recent research indicates that increasing precision in behavioural phenotypes using modern psychometric methods can increase signal within molecular genetics [32] as well within analysis of personality and cognitive correlates of psychopathology [25]. Such findings demonstrate that mathematical modelling can aid in validity studies of mental illnesses (see Forbes and colleagues [33] for a theoretical argument as well). In summary, the structure of mental illness and well-being in the general population is likely to be a multi-dimensional complex structure. Evaluating the structure of mental illness and well-being across a wide range of items may increase our understanding of mental health as well as increase the validity of behavioural signals identifying the aetiological components specifically underlying mental illness and wellness.

### The current study

Our analysis was conducted in three sections: evaluation of the structure of mental health using latent-trait methods, person-centred analysis of individuals with differing profiles of the mental health traits, and external validation of both the latent traits as well as the person-centred subgroups.

Many previous investigations that have indexed clinical symptom structure using item level data alone have only used measurements of anxiety and depression (e.g., [20,23]), with some studies additionally measuring psychotic experiences (e.g., [21,24]) and others a wider range of self and parent report domains (but not well-being; [22,27]). The evaluation of item level data combining mental well-being with items indexing clinical symptoms has often included only those relevant to depression and anxiety (e.g., [6–10]). Our aim was to characterise the common behavioural phenotypes of mental health across a wide-ranging spectrum of items denoting both mental well-being and illness within a community sample of 2228 individuals aged 14–24 years, which is an age group whose mental health structure has been understudied [34]. Measurement of both mental well-being and mental health involves a mixture of positively and negatively worded items. This carries potential confounding effects on factor structures [35,36] so we were careful to control for these wording effects within all of our modelling approaches in a manner previously suggested in the literature [37].

Although we evaluated a range of models, including single factor, correlated factor and second order solutions, we hypothesised that we would find a multidimensional behavioural structure in the youth population. We expected this would consist of a single general factor loading onto all items encompassing a construct ranging from positive well-being to negative illness items and denoting a mental perturbation expressed as distress (low through high). We also predicted there would be ‘distress independent’ specific factors that would not simply be left over variance of no consequence, but index specific unique characteristics of mental wellness and illness and also contribute to valid multidimensional behavioural patterning in the youth community at large. Therefore, our overall prediction was that there would be a bifactor structure, consisting of one general distress factor and distress independent factors. Our objective was to create a data driven behavioural phenotype that could be more closely associated with the underlying intermediate neurocognitive systems than single measurement constructs. This approach resonates with the RDoC initiative to more closely align neuroscience, cognition and behavioural phenotypes [31]. Several lines of evidence support the prediction of a bifactor structure. Previous findings have indicated a best fitting bifactor structure within mental illness [17–26], and a recent paper showed a similar structure when combining mental illness and mental well-being items in a sample of adults [10]. Additionally, a bifactor structure is suggested by literature showing high correlations between mental well-being and mental illness factors [6–9] and between externalising and internalising conditions (e.g., [14]). Finally, the vast literature detailing the high rates of comorbidity of mental illness in the general population supports the hypothesis of a higher order distress factor underlying many psychological diagnoses [38,39].

A second aim was to define subgroups of individuals with distinct combinations of both the general distress and specific distress independent factors. Since behaviour is multidimensional within as well as between individuals we hypothesised that deriving subgroups based on the aforementioned computed quantitative factorial levels would provide added value and greater validity for more mechanistic studies of mental health and illness going forward, as highlighted by recent reviews [34,40].

Finally, to provide a first test of validity of any new data driven factors and (factor based) subgroups we hypothesised that hazardous behaviours (self-harm, drinking and drug misuse) and self-reported experiences of treatment for mental ill health would discriminate between factors and derived classes. We predicted that those who engage in these hazardous behaviours or who have a history of treatment for mental ill health may have either altered factor scores or be more prevalent in certain subgroups. Specifically, hazardous behaviours would be associated with those factors relating to mental illness and subgroups defined by increased rates of general distress and/or specific factors related to mental illness.

## Methods

### Participants

The Neuroscience in Psychiatry Network (NSPN; nspn.org.uk) cohort consist of 2,257 volunteer participants aged between 14 and 25 recruited from Cambridgeshire (through the University of Cambridge) and London and surrounding areas (through University College London [UCL]). The recruitment of the sample included in this paper was from November 2012 to May 2014. Individuals who were currently taking part in a clinical trial of a medicinal or other therapeutic intervention were excluded. The main sampling framework comprised age-sex registers of 41 general practices (primary health care practices) recruited through the NIHR Clinical Research Network, and through 19 secondary schools/colleges/universities. Letters from the study team were sent in batches by the practices and schools to individuals in order to recruit into age and sex strata, as described below. Thus, we estimate that nearly all individuals who were eligible to participate were informed about the study opportunity. Additionally, our recruitment team made direct visits to secondary schools and colleges to support recruitments from this source. A minority (18.3%) were recruited directly through our website (www.nspn.org.uk). Written, informed consent was obtained for all participants over the age of 16 years. For participants under the age of 16, written informed assent was obtained for the participant and written informed consent from their parent/legal guardian.

Recruitment was stratified according to age and gender to give at least 200 males and females within each of the following five age categories [years;months]– 14;0 to 15;12, 16;0–17;12, 18;0–19;12, 20;0–21;12 and 22;0–24;12. One individual returned the study materials at 25;1 and was included. Consistent with previous studies [20], participants were included in our analysis if they filled in at least 85% of the total items (118 total items, minimum valid item count was 100) as well 85% of each individual measurement to ensure adequate data on all domains. There were a total of 29 individuals excluded based on this criterion. We next tested whether these individuals differed on the main measures investigated in the multivariate analysis. Due to the number of tests used, the significance value was lowered to *p* = .01. Significance levels between .01 and .05 were treated as marginally significant. The 29 individuals who did not meet the 85% criteria did not differ in age, gender, or total score of the MFQ (depressive symptoms), r-LOI (OCD symptoms), RSE (self-esteem), ABQ (antisocial behaviour), SPQ (psychotic symptoms), or WEMWBS (mental well-being). They had marginally higher total scores on the RCMAS (anxiety symptoms; *t*(2249) = -2.33, *p* = .02; M = 19.56, SD = 14.62 included; M = 26.54, SD = 11.85 excluded).

Thus, the study sample for this article is 2,228 individuals (54% girls). The mean age was 19 years (sd = 3 years). Sixty-one percent of the sample was recruited from Cambridgeshire. Differences across recruitment location in the final latent traits were evaluated. Several traits showed significant differences across location but most of these differences became non-significant when controlling for indices of multiple deprivation. This indicated that the majority of location differences were due to different distributions of multiple deprivation across Cambridgeshire and London. One latent trait (Specific Factor 5 –Mood) retained a significant location difference. With all subsequent analysis with this latent trait, recruitment location was included as a covariate. See the S1 Materials for a demographic summary of the sample.

Ethical approval was granted for this study by the NHS NRES Committee East of England—Cambridge Central (project ID 97546). The authors assert that all procedures contributing to this work comply with the ethical standards of the relevant national and institutional committees on human experimentation and with the Helsinki Declaration of 1975, as revised in 2008.

### Procedure

Members of the public were sent an invitation along with an expression of interest form via GP surgeries and local schools or colleges. Some participants responded to an advert or completed an expression of interest on the study website. Participants returned the completed expression of interest directly to the study team and were subsequently sent consent/assent forms (along with guardian consent forms for those under 16) as well as the study materials. Of those who expressed interest in possibly participating in the study and were sent study materials, 66% completed the completed consent forms and study materials; they received £10 in recompense for their time.

When compared on basic demographics, non-responders were more likely to be male (χ^2^(1) = 25.87, *p* < .001, V = .08), recruited from London (χ^2^(1) = 37.40, *p* < .001, V = .10) and slightly younger (*t*(2993.49) = 4.03, *p* < .001, *d* = .15; responders: M = 19;0 [years;months]; SD = 3;0; non-responders: M = 18;8; SD = 2;7). Responders were more likely to be white (χ^2^(1) = 63.08, *p* < .001, V = .13). When evaluated further, this difference was due to an over-representation in those who did not return the study materials of Asian, Black, and individuals who preferred not to indicate their ethnicity, and an over-representation of white individuals in those who did return the materials. There were no differences in those of mixed race or who ticked the “other” option. As more ethnic diversity was found in the London sample, we repeated the ethnicity comparison just within individuals recruited from London. Results were replicated with the exception that Asian participants were equally represented across responders and non-responders.

### Measures

All questionnaire measures used their original coding unless more than half of the items within each instrument had very sparse endorsement (< 1% of entire sample). In the latter instance, the sparse response categories were collapsed into adjacent categories.

#### Mental health measures

We wished to measure the common mental illnesses that emerge in the decade from 14 to 24 years where emotional and behavioural symptoms are the constituent components of the most commonly diagnosed disorders. The mental illness domains of interest to us were therefore anxiety and depressive disorders, conduct disorders, and obsessive compulsive and psychoses that emerge with discernible incident risk in adolescence and into the second decade of life [41]. In order to measure the whole of mental health, we included measures of positive mental health (self-esteem and well-being measures).

Thus, the following mental health domains were measured using self-reported measures covering the previous two weeks: Depression (33-item Moods and Feelings Questionnaire; MFQ; [42]); generalised anxiety that included measurements of physiological change, worry/oversensitivity, and social concerns (28-item Revised Children’s Manifest Anxiety Scale; RCMAS; [43]); obsessive compulsive items ([11-item Revised Leyton Obsessional Inventory; r-LOI; [44]); antisocial behaviours consisting of violating social norms, destructive behaviours, violence to people, lying and stealing (11-item Antisocial Behaviour Questionnaire; ABQ); self-esteem (10-item Rosenberg Self-Esteem Questionnaire; RSE; [45]); and mental well-being measuring positive evidence of current happiness, personal activity and personal achievement (14-item Warwick-Edinburgh Mental Well-Being Scale; WEMWBS; [46]).

We used a four category response version (never, mostly, sometimes, and always) for the RCMAS, r-LOI, ABQ, and RSE measurements where participants endorsed each item on how frequently they felt or behaved that way in the previous two weeks. Responses on the ABQ were converted to a “never” vs. “sometimes/mostly/always” binary response due to sparse endorsement of the mostly and always categories. The WEMWBS had five response categories (none of the time, rarely, some of the time, often, all of the time) with higher scores indicating higher mental well-being. All WEMWBS items were positively worded. Five RSE items were positive worded while the remainder were negative worded. No items were reversed for this analysis.

Participants completed the Schizotypal Personality Questionnaire (SPQ), a 74 item self-report questionnaire, measuring the items of the DSM-III diagnosis of Schizotypal Personality Disorder, including items related to psychotic-like experiences [47]. Response choices for all items were “present” or “absent”. As this study focused on measuring psychotic-like experiences, endorsement on the SPQ items was compared with positive ratings of psychotic-like experiences on the semi-structured PLIKS interview (PLIKSi) using a subsample of the total sample. PLIKSi measures 12 ‘core’ psychotic-like experiences [48]. Associations between SPQ items and the PLIKSi total symptoms as well as the hallucinations, delusions, and unusual perceptual experiences subscales were evaluated. Highly significant relationships with medium to large effect size were the criteria for inclusion. Additionally, consensus meetings between MCSC, IMG and PBJ agreed the retained items had face validity as psychotic items. Full details of this analysis can be found in the S1 Materials. The final items retained were SPQ 4, 9, 13, 28, 31, 40, 55, 60, 61, 63 and 64. See supplementary Table C in S1 Materials for item content for all measurements.

#### Discriminant validity measures

A self-report questionnaire measured past month alcohol, cannabis, and other UK illegal drugs (e.g., cocaine, heroin, MDMA/ecstasy, ketamine) use as well as non-suicidal self-injury (NSSI). Alcohol use was defined as never, sometimes or often/every day. Cannabis and other illegal drug use was defined as never or some use. NSSI was defined as never or once vs. twice or more, distinguishing those who had multiple occurrences of past month NSSI. Alcohol and drug use as well as NSSI were considered to be indicators of hazardous behaviours in the community; likely outcomes or consequences, rather than symptoms of these mental states directly. None of the items in the structural analysis overlapped with the hazardous behaviours used as discriminating variables. Current or past treatment for any emotional, behavioural or mental health problem was derived from parental reported (under 18) or self-reported (18 and over) difficulties. This was further broken down to measure specific treatment for depression.

### Analysis procedure

The latent trait analysis was based on the assumption that items would relate to each other in this population to reveal an underlying latent construct of mental health, across a positive and negative spectrum. This expectation is based on our prior work using item based analyses to elicit underlying latent structures, but to date we have only focused on characterising the illness or negative end of the latent construct [20,21]. There is, however, no clear-cut a priori theory to direct a specific analytic perspective using a combination of narrow band instruments. For this reason we chose to conduct analysis in a bottom up, data driven, and theory free manner in order to generate the latent structure underlying the interrelationships between the selected items. Additionally, we aimed to derive person-centred classes from the results of the structural factor analysis, which has previously been successful in identifying population based subgroups with distinctive probabilities for emotional and behavioural mental illnesses [49,50].

All data were processed through the Research Electronic Data Capture (REDCap) system [51]. Analysis was conducted in two stages, exploratory and confirmatory, with all indicators defined as categorical. The sample was split for these stages of analyses: half the data was used for the exploratory analysis while the second half used for confirmatory models. When fully confirmed, model fit was checked on the exploratory half (consistent with suggestions by Brown [52]) and was found to be similar to the confirmation half in all instances. Final confirmatory analysis was therefore conducted on the full sample. All items were scaled from 0 to the highest category (as specified in the materials section). The WLSMV (Weighed Least Square for categorical data Mean and Variance adjusted) estimator was used. All descriptive statistics, gender and age analysis and discriminate validity analyses were conducted within STATA MP 13.1 [53]. All multivariate analyses, unless otherwise specified, were conducted in MPlus version 7.2 [54].

#### Exploratory analysis

The exploratory factor analysis (EFA) for the first order models was conducted using the oblique promax rotation method, which is a methodology for evaluating correlated factor solutions. Previous work in our lab [20] investigating a subset the of the current indicators within a different sample evaluated all possible rotation methods and concluded that the promax rotation method produced the most interpretable solutions. We therefore chose this rotation method based on this previous work. An ESEM exploratory bifactor analysis was conducted using the bi-geomin rotation method. In line with the recommendation from Brown [52], the number of factors was determined through evaluation of the scree plot and eigenvalues as well as the model fit statistics for each potential factor solution. A more objective method, such as parallel analysis or minimal average partial tests, to determine factor number would have been preferable, but these options were not available in MPlus, nor was parallel analysis computationally possible in an alternative program (e.g., FACTOR; [55]).

#### Confirmatory analysis

All confirmatory analyses (first order and bifactor) allowed each item to be freely estimated with the factor variance set to 1. Thus, all factors had a mean of 0 and SD of 1. For the confirmatory factor analysis, the factor structure from the exploratory stage was implemented within a confirmatory framework. All loadings above .20 from the EFA (or .15 for the ESEM Exploratory Bifactor solution) were included on the factors, with the exception that any cross loading items below .25 were excluded unless both cross loading items were below .30. We utilised a relatively liberal cut off point for the EFA as we wanted to ensure we had as much information on each CFA factor as possible, with the aim of further refinement of the structure within the CFA phase. A methods factor was also included in all confirmatory analyses to account for the positive items within the entire item pool (five RSE and 14 WEMWBS items), as suggested by Wang and Lin [35] to control for spurious factors related to differences in the valence of item wording.

Items were added to factors as suggested by the modification indices, which estimate the improvement in model fit dependent on adding a specific item to one of the factors. Items were deleted from the factors if they were non-significant (p<0.05) or loaded below .25 for the correlated factor solutions and .20 for the Bifactor models. We note that others performing factor analyses of mental health data have applied equally stringent or even less stringent cut-offs [10,20,37]. Any item with cross loadings on multiple factors was evaluated. The lower loading was removed where one loading was below .30 (unless both were below .30). Cross loadings were also removed if there was a large discrepancy between the loadings (difference of .30). The final step was to consult the modification indices once more for the presence of any correlated errors between individual indicators. Correlated errors between similar items were either included or one item deleted, depending on the degree of similarity in what the items were measuring. Consensus meetings between authors MCSC, IMG and PBJ decided whether the correlated items indicated redundant items, where one item should be deleted, or related, but distinct items, where a correlated residual should be included. Further details of the confirmatory procedure can be found in the S1 Materials along with details of items which were deleted during the confirmation procedures.

In addition to an ESEM exploratory bifactor solutions (see S1 Materials for a summary of the final solution), we also investigated the Schmid-Leiman (SL) transformation of the CFA factor structures on the full sample. The ESEM exploratory bifactor explores the specific factors while estimating a general factor across all items. The SL transformation takes the original CFA factor structure as the specific factors and adds an additional general factor to the model across all items included in the final factor analysis. The SL transformation is computationally equivalent to adding one second order factor over all first order factors [52].

For all bifactor models, all loadings on the general factor had to be at least .30 on the general factor or they were dropped from the model [20]. The general and all specific factors were set to be uncorrelated with each other, creating an orthogonal factor structure.

#### Age and gender analysis

Regression analyses were conducted to determine how the latent traits differed by gender as well as developmental differences between the five different age categories. Analysis was done sequentially: firstly, with only main effects for gender and categorical age effects, then with a categorical age by gender interaction term. If the overall main effect of age category was significant, each comparison between the different age categories was conducted. For consistency, results were reported separately for males and females, even if the interaction was non-significant, indicating that the overall pattern was similar across males and females. A robust estimator was used when appropriate to control for unequal variances over the categories.

#### Latent profile analysis

The final model from the variable-centred latent trait analysis was evaluated in a person-centred latent profile analysis (LPA) using the whole sample. This analysis determined subgroups of individuals with similar patterning of profiles across all latent traits. The maximum likelihood robust (MLR) estimator was used within Mplus Version 7.2 [54]. As the goal was to combine the investigative strength of the bifactor approach with the pattern recognition of LPA, we opted for a two-stage procedure. Combining both the bifactor measurement model and the LPA analysis in a ML approach was computationally too intensive, and would require a change in the estimator in the bifactor model. Therefore, the latent traits were saved and then re-entered into the LPA analysis. Two to seven subgroups were evaluated through comparison of the AIC, BIC and BIC-SSA values as well as direct testing of lower order solutions using likelihood ratio tests (Vuong-Lo-Mendell-Rubin LRT, Lo-Mendell-Rubin adjusted LRT, and parametric bootstrapping LRT). Additionally, entropy was consulted, with solutions with higher entropy (and thus lower variability in category membership) being preferred. Gender and age were included as covariates on class membership in all models.

#### Discriminant validity

Ordered logistic or logistic regression analyses were used to predict the categorical variables (alcohol, cannabis, other illegal drug use, NSSI, MH treatment) jointly from all latent traits while also controlling for gender and age (as well as recruitment location when appropriate). A robust estimator was used when appropriate to control for unequal variances over the categories. For the LPA subgroups, the subgroup prevalence for the categorical variables was evaluated against the whole group distribution with a chi-square analysis. Due to the number of tests used, the significance value was lowered to *p* = .01. Significance levels between .01 and .05 were treated as marginally significant.

## Results

Following exploratory analysis, the scree plot and eigenvalues indicated that a four or five factor model could be perused. Therefore, these two models (alongside a one factor model for comparative purposes) were evaluated. Additionally, one second order and three bifactor models were fully investigated, meaning seven latent variable models were fully confirmed and evaluated, with model fit information presented in Table 1. In evaluating the seven solutions, careful consideration of the theoretical plausibility of all models was combined with evaluation of the absolute statistical model fit of each model to the data. The one factor model was not considered due to unacceptably low model fit, ruling out the possibility that a single factor is the best fit to mental health and wellness symptoms. The four and five factor models were considered and the absolute model fits were comparable, although the five factor model had a better absolute fit. However, the five factor model was also thought more theoretically plausible as the four factor model combined antisocial items with OCD and psychotic-like symptoms. No previous research to the authors’ knowledge has found that psychotic and OCD symptoms load on the same factor as antisocial items, except a general psychopathology factor [14,15,18]. Therefore, the five factor model, which split the “thought” items encapsulated in the OCD and psychotic-like items from the antisocial items, was considered more theoretically plausible.

**Table 1:** Latent trait modelling fit indices for fully confirmed models **.

Model	Chi Square	df	# parameters	CFI*	TLI*	RMSEA*	WRMR*
1 Factor Model	21847	5614	464	.933	.931	.036	2.222
1 Factor Model with sex, age, quadratic age and sex by age interaction covariates	21553	5823	464	.936	.934	.035	2.131
4 Factor Model	18082	5943	471	.951	.950	.030	1.914
4 Factor Model with sex, age, quadratic age and sex by age interaction covariates	17940	6258	485	.954	.952	.029	1.834
5 Factor Model	17096	5729	458	.953	.952	.030	1.893
5 Factor Model with sex, age, quadratic age and sex by age interaction covariates	17230	5924	474	.954	.953	.029	1.831
Second Order Internalising Factor on five factor solution	18183	5731	455	.949	.948	.031	1.984
S-L Bifactor (4 Specific Factors)	17503	5471	500	.951	.949	.031	1.883
S-L Bifactor (4 Specific Factors) with sex, age, quadratic age and sex by age interaction covariates	17223	5879	520	.954	.952	.029	1.804
***S-L Bifactor (5 Specific Factors)***	***15859***	***5350***	***510***	**.*956***	**.*955***	**.*030***	***1*.*795***
***S-L Bifactor (5 Specific Factors) with sex*, *age*, quadratic age *and sex by age interaction covariates***	***15567***	***5643***	***531***	**.*959***	**.*958***	**.*028***	***1*.*714***
Bifactor from ESEM Exploratory Bifactor Analysis	18104	5177	472	.946	.945	.033	1.999
Bifactor from ESEM with sex, age, quadratic age and sex by age interaction covariates	17905	5573	492	.950	.948	.032	1.924

*CFI = Comparative Fit Index; TLI = Tucker-Lewis Index; RMSEA = Root Mean Square Error of Approximation; WRMR = Weighted Root Mean Square Residual

**See Table B in S1 Materials for equivalent table detailing fit indices by age category and gender.

Similar rationale was applied in choosing the five factor SL bifactor model over the four factor SL bifactor model, as the inclusion of the general factor did not alter the structure of the specific factor in the four factor model which loaded antisocial, obsessive/compulsive as well as psychotic-like items on one factor. Additionally, the absolute model fit was stronger for the five factor SL model. Although the model fit differences between the four and five factor CFA and the related SL bifactor models did not differ substantially, there were unacceptably high correlations (r > .85) between the factors for the four and five factor CFA solutions, which is indicative of a general factor underlying all factors. We also tested whether these high correlations could be accounted for by a second order internalisation factor (accounting for the high correlations between conceptually relevant “internalising” first order factors) but this was not a good model fit and was not further evaluated. Thus, both CFA solutions were not preferred due to unacceptably high correlations and the four factor SL model was not preferred due to theoretically implausible specific factor structures. Finally, the bifactor model confirmed from the ESEM exploratory bifactor solution was not chosen as the model fit was lower than all other models (excepting the one factor model), with the CFI and TLI being below acceptable limits, although the factor structure was theoretically plausible. Therefore, the best fitting and most theoretically plausible model was the Schmid-Leiman bifactor transformation of the five-factor CFA solution, which is fully reported below. See Table 1 for detail of all model fits.

The final model consisted of one general factor and five independent specific factors. The general latent factor corresponded well to the notion of a general distress factor [20,21]. The general factor loaded negative items positively and positive items negatively, such that a higher score in the general factor is equivalent to higher distress and lower scores are related to lower distress and higher positive self-image. This supported our hypothesis that there would be a general extended latent trait factor across well-being as well as mental illness constructs. The specific factors are independent of this general distress latent construct. We tested the validity of these specific traits (see below) to determine if they contributed additional psychological information that aid in the discrimination of novel constructs or are merely redundant variance from the analysis of no clear cut value to detecting mentally well or ill individuals.

See Table C in the S1 Materials for the general and specific factor loadings (as well as the positive item methods factor) on each item as well as thresholds detailing how each item relates to the general distress and specific factors, (see footnote on Table C in S1 Materials for more details). Evaluation of the thresholds demonstrated that negative items generally provided good discrimination at only the high end of the distress and specific factors. This contrasted with positive items, which showed equal discrimination at the high (indicating low agreement with positive traits) as well as low end of the distress and specific factors (indicating high agreement with positive traits). These findings support our assertion that the inclusion of positive well-being items increases precision of the underlying construct of mental health.

Evaluating the severity thresholds also indicated that psychotic-like experience items, particularly those unrelated to social awareness, loaded very highly on the distress continuum, supporting Stochl et al.’s [21] contention that psychotic-like experiences are on the extreme end of a common mental distress continuum.

Specific factor 1 consisted of all 13 items from the well-being scale; we refer to this as a self-confidence factor. All items loaded positively, indicating that higher scores relate to higher self-confidence. Revealing this independent factor supports our speculation that there are independent positive constructs in the population that may aid identification of low risk sub populations of youth.

Specific factor 2 had nine items consisting of the antisocial behaviour items plus feeling like a bad person; we refer to this as an antisocial factor. All nine items loaded positively, indicating that higher scores related to increased antisocial behaviour.

Specific factor 3 had seven items measuring worry and being afraid all the time; a worry factor. All items loaded positively, indicating that higher scores related to increased levels of worry.

Specific factor 4 had 17 items measuring aberrant thinking, including obsessional/compulsive and psychotic-like experience items; an aberrant thought factor. All items loaded positively, indicating that higher scores related to increased levels of aberrant thoughts.

Specific factor 5 contained 30 indicators measuring unhappiness and anhedonia (absence of feelings) items, suicidality, negative self-comparison to others, as well as items relating to positive well-being; a mood factor. All negative items loaded positively, while positive self-esteem and well-being items loaded negatively, indicating that higher scores indicated increased mood problems.

**Fig 1:**
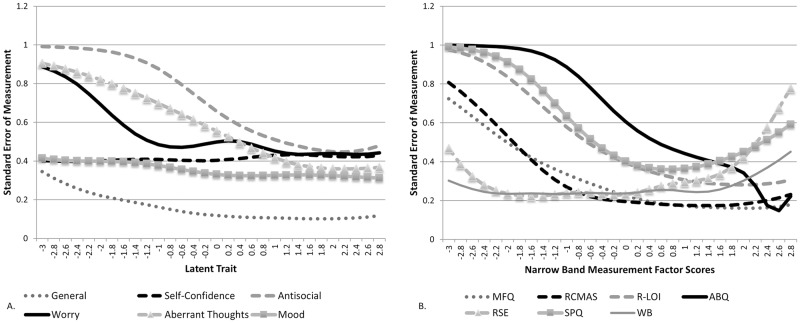
A. Standard Error of Measurement for the general factor and specific factors. B. Standard Error of Measurement for each Narrow Band measurement* Factor Score. *MFQ = Moods and Feelings Questionnaire; RCMAS = Revised Children’s Manifest Anxiety Scale; r-LOI = revised Leyton Obsessional Inventory; ABQ = Antisocial Behaviour Questionnaire; RSE = Rosenberg Self Esteem Scale; SPQ = Schizotypal Personality Questionnaire; WB = Warwick-Edinburgh Mental Well-Being Scale.

Fig 1A shows the standard error of measurement across all of the traits and demonstrates that all factors have high measurement precision across both high and low ends of each trait. However, there was a general pattern for lower measurement precision for the antisocial, worry and aberrant thought factors on the negative side of the trait. This pattern corresponds to traits with only negative items, whereas the self-confidence, mood and the general factor have consistent measurement precision across the entire trait, likely due to a mixture of both positive and negative mental health items. This supports our previous findings regarding the equal discrimination of positive items along the general distress continuum according to the severity thresholds.

For comparison purposes, we investigated the standard error of measurement of each of the individual narrow band instruments. Each instrument was turned into a factor with identical parameters as used above and showed a much more variable standard error of measurements across the latent traits (see Fig 1B). This result clearly indicates that similar levels of precision cannot be obtained across the entire range of a trait by simply using the narrow band measurements.

### Age and gender differences

**Fig 2:**
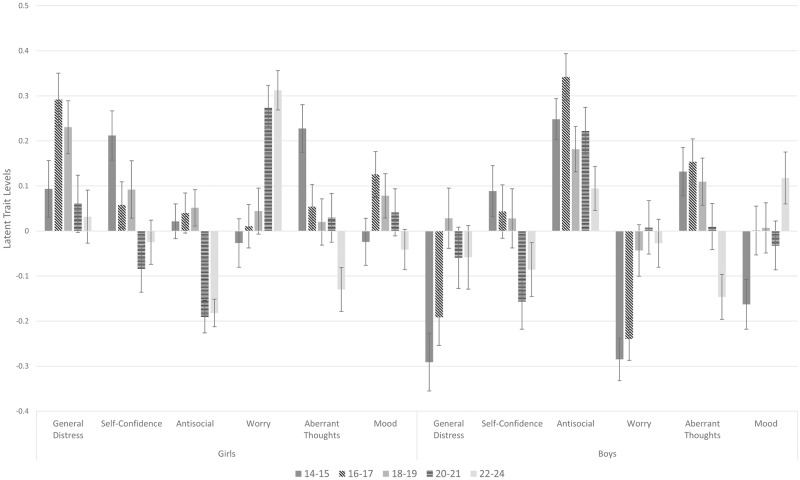
Bar chart showing general factor (distress), self-confidence, antisocial, worry, aberrant thoughts, and mood levels for girls and boys* at ages 14–15, 16–17, 18–19, 20–21 and 22–24. * Females: N = 224 for 14–15 year olds; N = 257 for 16–17 year olds; N = 234 for 18–19 year olds; N = 228 for 20–21 year olds; N = 261 for 22–24 year olds. Males: N = 199 for 14–15 year olds; N = 222 for 16–17 year olds; N = 200 for 18–19 year olds; N = 199 for 20–21 year olds; N = 204 for 22–24 year olds.

Fig 2 shows that there are both linear and non-linear age trends as well as gender effects, which vary between factors.

For the general factor (distress) girls had significantly higher overall scores than boys (β = .26, 95%CI(.18,.34,), *p* < .001, η^2^ = .02). This gender effect was qualified by an age grouping by gender interaction (*F*(4,2218) = 3.74; *p* < .005), indicating a different pattern throughout adolescence for girls and boys. For girls, there was a significant increase in general distress from early (age 14–15) to middle adolescence (16–17; *p* < .05), but no further significant differences to girls aged 18–19, 20–21 or 22–24 (all *p*’s > .10). General distress was elevated in middle adolescence (16–17) compared to early adulthood (20–21 and 22–24, *p*s < .01). Girls in late adolescence (18–19) also had higher general distress than in adulthood (22–24; *p* < .05). For boys there was a different pattern, with reduced general distress in early (14–15) and middle adolescence (16–17) compared to late adolescence (18–19; *p*s < .05). There was also significantly reduced distress in early adolescence (age 14–15) when compared to adulthood (20–21 and 22–24; *p*s < .05).

For the self-confidence specific factor there was no overall difference between girls and boys nor any interaction with age category, however there were differences across the age categories (*F*(4,2222) = 6.69; *p* < .001). For both girls and boys, there were higher levels of self-confidence in early adolescence (14–15) than adulthood (20–21 and 22–24; *p*s < .05). For both genders, there were also increased self-confidence in late adolescence (18–19) compared to early adulthood (20–21; *p*s < .05). For girls only, there was increased self-confidence in early (14–15) compared to middle adolescence (15–16; *p* < .05). For boys only, there was increased self-confidence in middle adolescence (15–16) compared to early adulthood (20–21; *p* < .05).

For the antisocial specific factor boys had significantly higher overall scores than girls (β = .27, 95%CI(.21,.33), *p* < .001, η^2^ = .04). This gender effect was qualified by an age grouping by gender interaction (*F*(4,2218) = 2.63; *p* < .05), indicating a different pattern throughout adolescence for girls and boys. For girls, there were no differences between early, middle and late adolescence, but each of these ages had higher antisocial traits than was found at age 20–21 (all *p* < .001). For boys, there were lower antisocial traits in adulthood (22–24) than in early (14–15) and middle (16–17) adolescence (*p*s < .05). There were also increased antisocial traits in middle (16–17) compared to late (18–19) adolescence (*p* < .05).

For the worry specific factor girls had significantly higher overall scores than boys, β = .24, 95%CI(.18,.31), *p* < .001, η^2^ = .02, but there was no interaction with age category, however there were differences across the age categories (*F*(4,2222) = 15.80; *p* < .001). For both girls and boys, there was a significant increase in worry traits from ages 14–15 and 16–17 to ages 20–21 and 22–24 (all *p*s < .005). Additionally, for girls only there was a significant increase from age 18–19 to age 20–21 (*p* < .005) and 22–24 (*p* < .001). For boys only, there was additionally significant increases in worry traits from ages 14–15 and 16–17 to age 18–19 (*p*s < .005).

For the aberrant thoughts specific factor there was no overall difference between girls and boys nor any interaction with age category, however there were differences across the age categories (*F*(4,2222) = 10.64; *p* < .001). For both girls and boys, there was a decrease in aberrant thought traits from all age periods when compared to age 22–24 (all *p*s < .05). For girls, there was an increased level of aberrant thoughts at age 14–15 when compared to ages 16–17, 18–19, and 20–21 (all *p*s < .05). For boys, there was significantly increased aberrant thoughts at age 16–17 when compared to age 20–21 (*p* < .05).

The mood specific factor did not differ overall between girls and boys, but there was a significant interaction between gender and age grouping (*F*(4,2217) = 2.59; *p* < .05). There was overall no main effect of age groupings for girls (*F*(4,1198) = 2.05; *p* = .09). For boys, there was a significant increased level of mood scores from early adolescence (14–15) when compared to ages 16–17, 18–19, and 22–24 (all *p*s < .05).

Full regression results of the specific age comparison can be found in Table D in the S1 Material. In addition to this analysis, the final model of all latent structures considered were run with just males, females and within the age groupings separately. A good fit the data from the SL 5 specific bifactor model was found for both genders and all age bands. See Supplementary Table B in S1 Materials for all fit indices.

### Latent profile analysis

Although individuals are frequently selected for investigation on the basis of high or low scores on single behavioural measures, multidimensional behavioural patterns are present in the majority of the population. Using a cut-off on any one measure alone will not capture the multidimensional nature of mental and behavioural states. We therefore investigated whether all the computed latent traits can be used to derive person-centred distinct subgroups. We used Latent Profile Analysis in order to reveal the best fitting number of subgroups with distinct patterns across all factors. We viewed this aspect of our analysis as proof of principle and hypothesis generating, aiming to reveal distinct sub populations based on the latent factor trait patterns in the sample.

**Fig 3:**
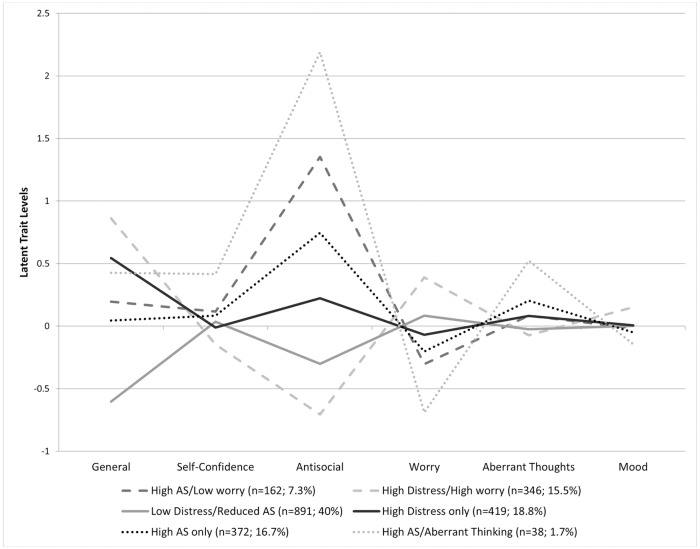
Latent trait levels for the six subgroups from the latent profile analysis.

The best fitting ‘class’ structure is summarised in Fig 3. See S1 Materials (p.35-36) for details on this analysis and Table 2 for the fit indices.

**Table 2:** Fit statistics of the LPA solutions for the sample.

Whole Sample	Log Likelihood	AIC	BIC	BICSSA	Entropy	VLMR test	LMR Adj	Bootstrap
Two classes	-15664.50	31370.99	31490.88	31424.16	0.78	--	--	<.001
Three classes	-15573.01	31206.02	31377.29	31281.97	0.61	0.001	0.001	<.001
Four classes	-15430.08	30938.15	31160.80	31036.89	0.75	0.017	0.017	<.001
Five classes	-15318.00	30732.00	31006.03	30853.52	0.80	0.029	0.030	<.001
**Six classes**	**-15228.92**	**30571.84**	**30897.24**	**30716.15**	**0.84**	**0.000**	**0.000**	**<.001**
Seven classes	-15237.11	30606.23	30983.01	30773.32	0.74	0.898	0.897	1.00

The general distress factor contributed to defining four subgroups while the (distress independent) specific worry factor helped define three population subgroups. The specific antisocial factor helped define five subgroups, but the self-confidence and aberrant thinking factors help define only one subgroup each. The specific mood factor did not vary above or below .25 SD in any subgroup, therefore only nominally contributed to discrimination of any subgroup.

Subgroup 1 (High Antisocial and Low Worry Subgroup) is defined by scores on two latent traits: a moderately high score (+1.25sd) on the specific antisocial factor with a lower score (-0.25sd) on the specific worry factor and consists of 7% of the sample, two thirds of which are boys.

Subgroup 2 (High Distress and High Worry Subgroup) is defined by a set of differential scores on three latent traits: high general distress (+.75sd) together with marginally higher worry (+0.25sd) and moderately lower (-0.5sd) antisocial factor scores. This subgroup consists of 16% of the sample of which three-quarters are girls.

Subgroup 3 (Low Distress and Reduced Antisocial Subgroup) is also defined by two latent trait scores: low general distress levels (-.5sd) and reduced antisocial traits (-.25sd) and no quantitative differences in any other latent trait. This subgroup consists of 40% of the sample with equal proportions of boys and girls.

Subgroup 4 (High Distress Only Subgroup) is defined by only one latent trait score: somewhat higher (+0.5sd) general distress levels. This subgroup consists of 19% of the sample, of whom three-fifths are girls.

Subgroup 5 (High Antisocial Only Subgroup) is also defined by only one latent trait score: a higher (+0.75sd) antisocial traits. This subgroup consists of 17% of the sample, of whom two-thirds are boys.

Finally, subgroup 6 (High Antisocial and Aberrant Thinking Subgroup) is defined by four latent trait scores: slightly higher on general distress and self-confidence factors (+0.25sd), very high antisocial (+2sd), moderately low worry (-0.5sd) and moderately high aberrant thinking (+0.5sd). This subgroup consists of 2% of the sample and four fifths are boys.

#### Age effects on class membership

The LPA analysis was repeated splitting by the sample at age 19 to evaluate whether there are age sensitive alterations in how the behavioural factors delineate subgroups of individuals. Age 19 was chosen due to the central location in the age distribution. Overall, there was a strikingly high correspondence between the solutions for those 18 and under with those 19 and over, which suggested no marked age effects in the latent profile structure. The main age difference was that the latent profile subgroups for those 19 and over showed much more differentiation in the self-confidence and aberrant thoughts specific factors and slightly less differentiation in the antisocial specific factors. In particular, the antisocial factor was reduced in Subgroup 5. This indicates that by adulthood the self-confidence and aberrant thoughts specific factors may become increasingly important in differentiating subgroups of individuals, while the influence of the antisocial specific factor reduces. See supplemental materials for details of the age split analysis.

### Discriminant validity

A summary of the findings from the initial discriminant validity analysis of the general distress and specific factors and LPA subgroups are shown in Tables 3 and 4.

**Table 3:** Details of how the general distress factor and all five specific factors relate to hazardous behaviour (alcohol and drug use, NSSI) and past/current treatment for mental ill health.

*Latent Traits*	*General (distress)*	*Self-Confidence*	*Antisocial*	*Worry*	*Aberrant Thoughts*	*Mood*	*N (%)*
**Alcohol Use**							
1. Never	.01 (1.01)	.06 (.89)	.03 (.67)	-.02 (.80)	.12 (.75)	.03 (.81)	752 (34.0%)
2. Sometimes	-.002 (.93)	-.01 (.82)	.06 (.65)	.05 (.76)	.02 (.79)	.002 (.77)	1213 (54.7%)
3. Often/Every Day	.18 (.01)	.05 (.93)	.27 (.77)	-.03 (.75)	-.08 (.74)	.02 (.81)	251 (11.3%)
Comparisons	3 > 1 = 2	ns	3>2>1	ns	3 < 1	ns	
**Cannabis Use**							
Never	-.02 (.95)	.02 (.85)	.02 (.64)	.02 (.79)	.04 (.77)	.01 (.78)	1937 (87.3%)
Some Use	.29 (.95)	.01 (.91)	.47 (.77)	-.05 (.72)	.08 (.80)	.04 (.78)	283 (12.7%)
β (95% CI)^1^	.34 (.20-.48)***	-.03 (-.19-.13)	.90 (.72–1.08)***	.08 (-.08-.24)	-.01 (-.18-.17)	.09 (-.07-.26)	
**Illegal Drug Use**							
Never	.01 (.95)	.01 (.86)	.06 (.66)	.02 (.78)	.04 (.77)	.02 (.79)	2125 (95.8%)
Some Use	.36 (1.00)	.14 (.90)	.47 (.85)	-.05 (.74)	.001 (.77)	-.07 (.67)	94 (4.2%)
β (95% CI)^1^	.37 (.14-.60)**	.16 (-.09-.41)	.83 (.54–1.11)***	-.02 (-.28-.25)	-.17 (-.47–13)	-.15 (-.40–11)	
**NSSI**							
Never or once	-.06 (.90)	.03 (.86)	.06 (.66)	.02 (.78)	.04 (.77)	-.01 (.77)	2056 (92.8%)
more than twice	1.14 (.92)	-.15 (.82)	.17 (.78)	-.09 (.78)	.08 (.79)	.33 (.84)	160 (7.2%)
β (95% CI)^1^	1.43 (1.21–1.65)***	-.22 (-.42–-.03)±	.36 (.11-.61)*	-.13 (-.36-.09)	.07 (-.17-.31)	.51 (.28-.74)***	
**Past/Current Treatment for Mental Ill Health**							
**Any treatment**							
None	-.09 (.91)	.04 (.86)	.09 (.67)	-.01 (.76)	.05 (.76)	-.01 (.78)	1875 (84.7%)
Treatment	.64 (.98)	-.11 (.84)	-.04 (.69)	.13 (.83)	.01 (.86)	.13 (.79)	338 (15.3%)
β (95% CI)^1^	.83 (.69-.97)***	-.13 (-.28-.02)	-.16 (-.36-.032)	.09(-.06-.26)	.05 (-.12-.21)	.12 (-.04-.28)	
**Depression Treatment**							
None	-.05 (.92)	.04 (.86)	.09 (.67)	.001 (.77)	.06 (.77)	-.01 (.78)	2025 (91.5%)
Treatment	.81 (1.02)	-.19 (.85)	-.11 (.68)	.12 (.82)	-.14 (.86)	.25 (.75)	188 (8.5%)
β (95% CI)^1^	.97 (.79–1.15)***	-.16 (-.36-.04)	-.16 (-.43-.10)	-.03 (-.25-.18)	-.16 (-.37-.06)	.28 (.07-.49)*	

^1^ β values and 95% CI measuring the differences in the general distress and specific factors across the binary categorical variables.

* *p* < .01;

** *p* < .005;

*** *p* < .001

**Table 4:** Details of how the six subgroups relate to the general distress factor and five specific factors, hazardous behaviour (alcohol and drug use, NSSI) and past/current treatment for mental ill health.

*LPA Subgroups*	*Whole Group (N = 2228)*	*SG1/High Antisocial and Low Worry (N = 162)*	*SG2/High Distress and High Worry (N = 346)*	*SG3/Low Distress and Reduced Antisocial (N = 891)*	*SG4/High Distress Only (N = 419)*	*SG5/High Antisocial Only (N = 372)*	*SG6/High Antisocial and Aberrant Thinking (N = 38)*
**Factor Scores**							
General Distress	--	0.20 (.86)	0.86 (.66)	-0.60 (.75)	0.54 (.80)	0.05 (.82)	0.43 (.82
Self-Confidence	--	0.12 (.83)	-.15 (.86)	0.04 (.83)	-0.01 (.85)	0.08 (.87)	0.42 (1.14)
Antisocial	--	1.35 (.19)	-0.71 (.19)	-0.30 (.14)	0.22 (.16)	0.75 (.17)	2.19 (.25)
Worry	--	-0.30 (.72)	0.39 (.87)	0.08 (.71)	-0.07 (.76)	-0.20 (.71)	-0.69 (.64)
Aberrant Thoughts	--	0.08 (.73)	-0.07 (.84)	-0.02 (.74)	0.08 (.77)	0.20 (.75)	0.52 (.87)
Mood	--	-0.02 (.83)	0.15 (.85)	0.003 (.73)	0.01 (.77)	-0.05 (.81)	-0.14 (.83)
**Alcohol Use**^1^							
Sometimes	55% (1213)	54% (88)	56% (192)**	56% (493)	52% (217)	57% (212)	29% (11)***
Often/Every Day	11% (251)	16% (26)	6% (19)**	10% (89)	15% (61)	11% (42)	37% (14)***
**Cannabis Use**^1^							
Some Use*	13% (283)	28% (46)***	7% (25)**	7% (63)***	14% (59)	20% (76)***	37% (14)***
**Illegal Drug Use**^1^							
Some Use*	4% (94)	9% (15)**	2% (7)	3% (25)	5% (19)	5% (20)	21% (8)**
**NSSI**^1^							
more than twice	7% (160)	9% (14)	14% (47)***	2% (16)***	11% (46)±	9% (32)	16% (6)
**Past/Current Treatment for Mental Ill Health**^1^							
Any treatment	15% (338)	13% (20)	30% (101)***	11% (92)***	17% (69)	13% (50)	16% (6)
Depression Treatment	9% (188)	7% (11)	20% (70)***	5% (45)**	8% (35)	6% (24)	8% (3)

^±^ *p* < .05

^&^ *p* > .01;

* *p* < .01;

** *p* < .005;

*** *p* < .001

^1^ For the LPA subgroups, the distribution for each subgroup was compared to the distribution for the whole group. The reported significance relates to chi square or Fisher’s Exact tests.

#### Hazardous behaviours

Elevations in both the general distress factor and the specific antisocial factor were independently associated with all four hazardous behaviours (increased alcohol, cannabis, illegal drug use and non-suicidal self-injury [NSSI]). Elevations in the specific mood factor were only associated with the occurrence of NSSI. In contrast, the specific factors worry, aberrant thoughts and self-confidence were not related to any hazardous behaviours.

Subgroups with increased levels of antisocial traits (High Antisocial and Low Worry, High Antisocial Only, and High Antisocial and Aberrant Thinking Subgroups) were the only subgroups to have increased rates of both cannabis and other illegal drug use. Alcohol consumption was also increased in the High Antisocial and Aberrant Thinking Subgroup.

Interestingly, increased NSSI was associated with the High Distress and High Worry subgroup while the Low Distress and Reduced Antisocial subgroup was associated with lower reporting of this behaviour. In addition, the Low Distress and Reduced Antisocial subgroup was associated with reduced use of cannabis and illegal drug use. Furthermore, the High Distress Only Subgroup showed normative levels on all hazardous behaviours.

#### Treatment for mental health difficulties

Higher scores on general distress were associated with treatment for mental ill health. This factor and the specific mood factor were both associated with treatment specifically for clinical depression. There were no other associates with the other specific factors.

Amongst the subgroups, members of the High Distress and High Worry Subgroup were twice as likely to have experienced any treatment for mental ill health as well as twice as likely to have experienced treatment for depression.

Conversely, members of the Low Distress and Reduced Antisocial Subgroup were less likely to have experienced any type of treatment for mental ill health. It is of note that the High Distress Only Subgroup showed normative levels (average for the entire sample) of treatment for mental ill health, even though this subgroup was characterised by high general distress only. The remaining subgroups also showed normative levels.

Overall, there is a greater association signal with treatment for mental ill health as well as hazardous behaviours for subgroups containing patterns of traits rather than single traits alone. This confirms the value of delineating the multidimensionality of behavioural repertoire in the population at large to appreciate the nature of associated risks.

## Discussion

To our knowledge this is the first report to use both multivariate variable and person-centred strategies to evaluate the organisation of current mental health characteristics, encompassing a wide range of mental well-being and mental illness domains, in an adolescent and young adult population-based volunteer sample. This data-driven approach has revealed a novel latent structure of thoughts, feelings and behaviours, as well as characterising new population subgroups based on the patterning of their factor profiles.

Consistent with previous research finding a separation of internalising (usually measuring anxiety and depression symptoms) and externalising (usually measuring antisocial behaviours) symptoms in the structure of mental illness was revealed: internalising (general distress and specific mood and worry factors) and externalising (the specific antisocial factor) variable-centred factors emerged. However, the findings also indicate a different organisation to that reported in prior population based item/symptom and diagnosis factor studies (e.g., [13]). Previous studies had suggested that the behavioural structure consisted of only these two components (internalising/externalising), which were orthogonal to each other. Our results reiterate the importance of internalising and externalising dimensions, while replicating previous findings of a general distress or psychopathology factor underpinning both [18–26].

Many of the factors delineating the commonly measured depression and anxiety traits were subsumed entirely under the general distress psychopathology factor. Indeed, 33 of the 37 items loading only on the general distress factor came from the depression and anxiety measurements, and nearly all the items relating to somatic features (e.g., moved and walked more slowly than usual, didn’t sleep as well as usual) loaded only on the general distress factor. From this we draw several conclusions. First, somatic features may be a common feature across many psychiatric conditions, which indeed are reflected in the existing diagnostic criteria. Second, many items included in common measurements of anxiety and depression seem to be tapping into the general distress underlying both conditions. The items loading both on specific factors as well as the general factor were more personal, self-comparative to others, and related directly to mood, anhedonia and worry symptoms, indicating that these items may be more specific to distinct mental health conditions. These findings may be of relevance for the interpretation, construction and usability of these single domain measurements.

The idea that mental health and well-being might be best characterised by one general factor with specific factors relating to more unique aspects of either mental well-being or mental illness has important consequences for the overall measurement of mental health, with possible implications for the diagnosis of mental illness. The multidimensional nature of the bifactor solution need not necessarily indicate that we must measure all domains or items in order to accurately measure the general distress factor [56]. Based on the current results, we suggest a good measure of general distress should include a combination of positive and negative mental health items, but need not include all dimensions measured in this paper. As Reise argues as well, the bifactor model also allows for the measurement of subscales independent of the general distress factor, which is more difficult to model in the related hierarchical factor structure (with first and second order factors [56]). Thus, the bifactor nature of the structure endorsed here, as well as in numerous other papers [20–24], has important implications for the recognition of both mental well-being and mental illness. Summary of subscales or of the general factor total score are possible for both future research studies as well potentially within clinical populations where the computationally intensive methods utilised here are not feasible.

The person-centred analysis reveals for the first time a definable patterning of factor scores resulting in distinctive subgroups based on multidimensional behavioural characteristics in youth. This population heterogeneity may help to explain marked individual differences in symptom profiles and clinical outcome within a psychiatric syndrome. For example, clinical syndromes, such as major depression, are derived from 12 symptoms in differing patterns and therefore may well arise from distinctive population subgroups with putatively different aetiologies, as we have previously suggested [49].

While many of the subgroups were differentiated on many different factors, the specific factor measuring antisocial behaviour seemed to have a distinctive level in each subgroup. This trait had the largest mean variation in the subgroups, with the highest subgroup’s mean 2 SD above the sample average (although this subgroup only accounted for 2% of the sample). This perhaps reflects the distinctive pattern of externalising behaviour in adolescence and how individual propensities towards antisocial behaviour vary widely on different factors [57]. It may also be that differing levels of antisocial behaviour are highly predictive of other, more internalising traits. It is of note when the LPA analysis was replicated separately within the adolescent and young adult subsamples, the antisocial specific factor remained highly differential in the adolescent subgroups, but less so in the young adult subgroups. Further research evaluating the LPA structure in this sample is needed to further illuminate why the antisocial specific factors discriminates between the subgroups to such a high degree.

### Added value of person-centred analyses

Revealing a common or general distress factor provides an explanation for covariance and trans-diagnostic effects found for self-report measures of ostensibly different symptom profiles as well as the commonly reported comorbidity across differing psychiatric diagnoses [18,19,22,49,58,59]. Such a factor subserving numerous clinical phenotypes may also explain why cognitive vulnerabilities, such as those revealed using computerised tasks, are associated with higher scores on multiple narrow band behavioural scales when completed concurrently [58]. However, it seems that the person-centred approach may improve the precision and validity of individual latent factors. For example, examining the general distress factor scores suggests higher scoring individuals are more likely to report increased hazardous behaviours and mental health service use. However, the High Distress Only subgroup, with median levels for all other factors, was not associated with either hazardous behaviours or treatment for mental ill health. These findings may help to explain why screening for general distress in either primary healthcare or within specific medical conditions is an inefficient way of finding and treating individuals who are in need of psychiatric care as it is likely that many higher scorers on such scales have low prevalence for mental illness [60–62]. Of course, it may be that increased distress alone may act as a generic risk factor for future mental health issues for a subset of individuals, although it is not indicative of a current or past disorder. Longitudinal studies based on this multidimensional structure and distinct subgroups are required to answer these important prognostic questions.

### The discovery of a potential low mental illness risk subgroup

The inclusion of positive well-being items is likely to have made two important contributions to our results. First, there was increased precision of factor measurement across the entire trait in both the general distress and specific factors, indicating that positive items not only predict low distress but the absence of endorsement predicts high distress. Negative items only predict high distress, as their absence was not predictive of low distress. Second, we have revealed for the first time a subpopulation with very low risk of mental illness: the Low Distress and Reduced Antisocial subgroup, comprising 40% of our sample. Revealing ‘wellness’ subgroups to date have mainly been based on the absence of a positive response to illness items, which, as mentioned above, is empirically less satisfactory and valid than looking at positive endorsement on positive items. This subgroup with low mental illness risk is characterised by low general distress together with reduced antisocial tendencies and the population mean of all other factors. As preliminary evidence we note that this subgroup shows reduced levels of cannabis use, reduced history of engaging in non-suicidal self-harm and reduced history of treatment for mental ill health. The identification of this potential low mental illness risk subgroup might also provide new opportunities for investigating conceptually complex notions, such as the underpinning of a resilient human brain and mind [63]. This subgroup also provides new opportunities to reveal distinctions in the brain architecture between those at low risk and the mentally ill [64].

### Age and sex effects and their implications for behavioural theory

We found gender and age differences within the general distress factor that replicates the common pattern in the depression literature of a mid-adolescent apex followed by a slow decline for girls and stability for boys [65]. This finding, while consistent with depression literature, does not replicate the absence of gender differences in previous studies of general distress or psychopathology factors observed in childhood and adulthood samples [18,23,24]. This may be due to the age range of this sample being 14 to 24 years when gender differences are most likely to occur in depression. However, the multidimensional structure does reveal some novel age and sex effects that may have a bearing on the incident risk of mental illnesses into the third decade.

#### Reflective worry and cognitive maturation

The specific distress independent worry factor indexes an age increase in factor scores for both sexes consistent with the emergence of a more reflective top down cognitive process. For example, this could be a goal directed or model based system, available to allow flexible adaptation to environmental challenges [66].

The added value of the subgroup analysis is further illustrated when considering the potential adaptive role of (reflective) worry. Low worry in the High Antisocial and Low Worry and High Antisocial and Aberrant Thinking subgroups in combination with high antisocial and/or general distress factor scores may index insufficient deployment of top down cognitive abilities, which may help explain the elevated magnitude of association with hazardous behaviours in these subgroups. This is supported by evaluation of the High Distress and Worry subgroup, which was indexed by high worry and distress and lowered antisocial traits. This subgroup showed reduced levels of alcohol and cannabis use, but increased rates of NSSI and mental illness treatment. These findings indicate that increased reflective worry may either be an additive risk factor (indicating higher levels of negative or maladaptive worries) or an insufficient reflective ability to offset very high general distress scores. In all, the specific worry factor may index, within the subgroups, either insufficient cognitive control when decreased or maladaptive responses when increased, particularly in combination with increased general distress. While these musings are speculative and need further evaluation, we can be clear regarding the association between antisocial traits and this worry factor—higher antisocial traits are related to lower worry across all subgroups, while decreased antisocial traits relate to higher levels of worry.

#### Specific mood, self-confidence and aberrant thought factors

The distress independent specific mood factor shows a further distinctive age and sex pattern not previously revealed in prior studies. This factor shows an increase in age for boys not revealed for girls. Taking the analyses as a whole and noting the associations between elevated factor scores on this mood factor with self-harm and treatment for mental ill health we conclude that elevated mood scores indexes maladaptive mood change.

It is striking that this mood factor does not contribute to defining any subgroups. This suggests that elevated mood factor scores alone are not sufficient to lead to the emergence of a clinical syndrome. However, the specific mood factor was striking in that it contained measurements of mood related to depression, yet we did not find the normal gender differences and only evidenced developmental changes for boys and not girls. Thus, this specific component may reflect more a stable “depressogenic” characteristic which is emerging in boys, but consistently present at the same level throughout our developmental window for girls. Given the lack of subgroup differentiation, it may be that the more state-like components of depression are expressed through the general distress factor. The more stable component of depression may be measured in the specific mood factor. This may relate to cognitive biasing mechanisms associated with depressive disorders, potentially promoting negative evaluations of self, as these items featured heavily on this specific factor. Future research investigating longitudinal differences within this multivariate structure will seek to illuminate the psychological implications of this specific factor further [67].

Finally, the specific factors for self-confidence and aberrant thoughts both showed a decline with age, which was not sex differentiated. Furthermore, these factors alone are not associated with any increases in hazardous behaviours or treatment for mental ill health. The precise psychological implications of these traits are, however, not clear-cut. Their role in identifying factor based sub-groups is confined to the smallest and most deviant group where both are increased. It is not readily apparent what their function may be in this subgroup. More work on the self-confidence and aberrant thought factor is required to understand their potential contribution to mental health and illness in the population.

### Limitations

This study is not without limitations. First, the measurement of mental illness is restricted to most, but not all, common domains likely to encompass the emergence of psychiatric disorders in the second decade of life. For example, we did not have our sample report specifically on mania or specific eating disorder items, such as body image distortion. Unfortunately, practical considerations limited the domains we were able to measure, as each participant filled in the questionnaires used in the study in addition to other questionnaires measuring different domains of interest (personality and environmental influences). Second, we cannot be sure these factors would be correct for younger or older populations. Indeed, our split age LPA indicates that the self-confidence and aberrant thoughts factors may be increasingly important in early adulthood, indicating a slight age shift in the patterning of the factors. Furthermore, natural variations in our sample (age split and gender) were only tested for measurement invariance in the most basic manner, by evaluating the model in each individual subgroup. While these results indicated a good model fit across both genders and all age bands, full measurement invariance was not conducted due to concerns about adding additional complexities to an already complex paper. Future research could investigate and validate the measurement invariance across the genders and age categories within this sample.

Additionally, there has been debate in the literature regarding the meaning of measurements and overarching constructs (such as mental wellness and illness) when loading on an overarching factor, such as our general distress factor. While some authors have concluded that numerous measurements that load strongly on a general distress factor indicate that the individual measurements are overlapping [10], we take a different approach. While we acknowledge that while many psychological well-being and mental illness questionnaires do tap into a common underlying construct (which we term general distress), we note that the validity and utility of the questionnaires themselves should not be necessarily questioned or regarded as “straw men” measuring the same underlying construct. Indeed, the variety of questionnaires used in the current analysis, as well as the specificity found within our specific factors, argues against any explanation that the general distress factor is simply reflecting a lack of differentiation within the questionnaires evaluated. Rather, we believe that we are capturing the common variance across both mental wellness and illness (and the associated questionnaires) while allowing specific, more unique variance to be captured by the specific factors, which often (but not always) reflect the underlying questionnaires used. No single measure was entirely subsumed under the general distress factor, although we have already discussed that the larger proportion of anxiety and depression items only related to the general distress factor. This indicates that these common mental states inherently have a larger component of cross-diagnostic general distress. Additionally, recent research within a large twin sample has found that the general factor (with mental illness indicators) was moderately heritable, which the authors indicate argues against the idea that the general factor is a methodological artefact [68].

There is a wider debate on the usefulness and interpretation of these specific factors. As reported by Chen et al. [37] specific factors can provide additional and useful information above and beyond the general factor, which was seen in our discriminate validity findings. Indeed, Waldman and colleagues [68] found varying contributions from genetics and the environment across specific factors, indicating good signal within specific factors. In the current study, all specific factors, with the exception of the self-confidence and worry specific factors, showed some associations with hazardous behaviours or treatment for mental ill health (or both). The self-confidence and worry specific factors instead showed discrimination within the subgroups as well as clear development trends. Thus, our five specific factors appear to be useful constructs above and apart from the general factor. We are currently further validating and extending this work, specifically within longitudinal analyses, which will help clarify the precise meaning and information contained within these specific factors.

Another limitation of this study is the possibility that some of our participants may have been biologically related. This was investigated and we found 84 known sibling pairs in the sample and four households with three siblings, so in total there were at least 180 individuals with some relatedness to other participants. However, our sampling framework allowed us to identify relatedness only on the basis of participant name and address. We conducted a control analyses that indicated the results were replicated when controlling for the known relatedness within the sample. Therefore, this limitation in our sampling framework does not appear to have had a substantial influence on our results.

Both a strength and limitation of the study is the sample size. The sample size is more than adequate for the latent trait and LPA analysis, but it remains an empirical question whether at the population level there are even more unique subgroups of individuals that this study’s sample size (and recruitment parameters) do not allow us to delineate. A strength of this study, however, was the inclusion of self-esteem and well-being items, which added a positive dimension to the consideration of the structure of mental health and allowed for the measurement of both well and ill health. Finally, our study is confined to cross sectional analysis and therefore requires replication and an examination of prognostic and predictive validity of the traits and sub groups described. In this regard, the findings represent a proof of principle investigation of mental health structure in the youth population at large.

### Conclusion

In summary, the use of two formal multivariate mathematical models reveals a unique structural organisation and patterning of self-reported mood, feelings, thoughts and behaviours in a youth population at large. The multidimensional bifactor solution both replicates previous findings [e.g., 10,20,23,24,27] regarding the structure of mental health as well as extends the literature by using a wide range of indicators of both mental illness and mental well-being. Further to evaluating the structure of these symptoms, we have established differing subgroups of individuals across the variable-centred factors. The combination of both methodologies allows a better context for understanding and evaluating these factors, and in particular has allowed an identification of a low risk subgroup with characteristics that may index resilience and a potential differential role of the specific worry factor to hazardous behaviours and mental health treatment, dependent on the levels of the general distress and other specific factors. The combination of variable-centred and person-centred approaches is suggested for future research.

## Supporting information

S1 Materials Provides additional information regarding alternative factor structures evaluated but not retained, further information regarding analytic procedure, and more detailed statistical analysis. (DOCX)
