# Supplementary material for: Characterising the latent structure and organisation of self-reported thoughts, feelings and behaviours in adolescents and young adults
Source: PLoS One. 2017 Apr 12;12(4):e0175381. doi: 10.1371/journal.pone.0175381 (PMC5389661; doi:10.1371/journal.pone.0175381)
Supplement: S1 Materials — (DOCX) [file pone.0175381.s001.docx]

**Supplementary Materials**

Contents

[List of Tables 2](#_Toc472262486)

[List of Figures 2](#_Toc472262487)

[Demographics of the Sample 3](#_Toc472262488)

[Comparison of the SPQ items to Psychotic Experiences 4](#_Toc472262489)

[Details of Discriminant Validity Coding 5](#_Toc472262490)

[Fit indices by gender and age category 6](#_Toc472262491)

[Details of the final bifactor model: SL 5 specific bifactor model 8](#_Toc472262492)

[Further Details on the Analysis Procedure 22](#_Toc472262493)

[Further details on the Exploratory Factor Analysis and Bi-Geomin ESEM 22](#_Toc472262494)

[Further details on the Confirmatory Factor Analysis 23](#_Toc472262495)

[Details of items deleted from all fully confirmed models 26](#_Toc472262496)

[One factor first order model. 26](#_Toc472262497)

[Four factor first order model. 26](#_Toc472262498)

[Five factor first order model. 26](#_Toc472262499)

[ESEM Exploratory and Confirmatory Bifactor analysis. 27](#_Toc472262500)

[Schmid-Leiman Bifactor Transformation of Four Factor CFA. 27](#_Toc472262501)

[Schmid-Leiman Bifactor transformation of five factor CFA. 27](#_Toc472262502)

[Latent Structural Analysis of the First Order Models: Further Details 28](#_Toc472262503)

[One factor first order model. 28](#_Toc472262504)

[Four factor first order model. 28](#_Toc472262505)

[Five factor first order model. 29](#_Toc472262506)

[Second order internalisation factor. 30](#_Toc472262507)

[ESEM Exploratory and Confirmatory Bifactor analysis 30](#_Toc472262508)

[Schmid-Leiman Bifactor Transformation of Four Factor CFA. 31](#_Toc472262509)

[Age and Gender Differences: Full results 32](#_Toc472262510)

[Latent Profile Analysis: Further details 35](#_Toc472262511)

[Latent profile analysis: Five class solution for those 18 and under 38](#_Toc472262512)

[Latent profile analysis: Six class solution for those 19 and over 39](#_Toc472262513)

[References 41](#_Toc472262514)

# List of Tables

[Table A. Sociodemographic characteristics of this included participants (n = 2,228) 3](#_Toc478984427)

[Table B. Latent trait modelling fit indices by gender and age category. 6](#_Toc478984428)

[Table C. Loadings for the general factor and five specific factors from the SL transformation of the 5 factor CFA. Severity thresholds^1^ for each item also given. 8](#_Toc478984429)

[Table D. Full results of gender and age category differences. 31](#_Toc478984430)

[Table E. Fit statistics of the LPA solutions for only those 18 and under and only those 19 and over. 37](#_Toc478984431)

[Table F. Gender distribution and average age for all six subgroups. 38](#_Toc478984432)

# List of Figures

[Fig A. Latent Trait Levels for the six subgroup latent profile analysis for participants aged 18 and under. 39](#_Toc478984401)

[Fig B. Latent Trait Levels for the six subgroup latent profile analysis for participants aged 19 and over. 40](#_Toc478984402)

# Demographics of the Sample

Table A. Sociodemographic characteristics of this included participants (n = 2,228)

|  | 14/15  N = 423 | 16/17  N = 479 | 18/19  N = 434 | 20/21  N = 427 | 22-24  N = 465 |
| --- | --- | --- | --- | --- | --- |
| Percentage (N) Female | 53% (224) | 54% (257) | 54% (234) | 53% (228) | 56% (261) |
| Mean age (SD) | 15.03 (6.75 months) | 17.12 (6.31 months) | 18.85 (7.11 months) | 20.95 (6.90 months) | 23.46 (10.34 months) |
| Percentage Cambridgeshire | 62% (261) | 58% (280) | 59% (255) | 62% (265) | 64% (299) |
| Ethnicity |  |  |  |  |  |
| White | 81.32% | 73.28% | 76.73% | 81.03% | 83.44% |
| Asian | 5.44% | 11.06% | 9.91% | 7.03% | 6.02% |
| Mixed | 7.33% | 6.89% | 4.84% | 4.45% | 4.30% |
| Black | 3.07% | 4.80% | 4.61% | 1.64% | 2.37% |
| Other | 1.65% | 1.46% | 2.53% | 2.58% | 1.51% |
| Prefer not to say | 1.18% | 2.51% | 1.38% | 3.28% | 2.37% |

# Comparison of the SPQ items to Psychotic Experiences

All items from the SPQ were compared to endorsement of psychotic experiences as measured by the PLIKSi. The PLIKSi is a semi-structured interview that measure 12 ‘core’ psychotic experiences (Horwood et al., 2008).

Three subdomains comprise: hallucinations (visual and auditory), delusions (e.g. being spied upon, persecution, telepathy, grandiose ability), and unusual perceptual experiences (e.g. thoughts being broadcast). All these experiences had a core question, elaborated with additional questions when necessary. Seven core questions came from the Diagnostic Interview Schedule for Children-IV (Shaffer, Fisher, Lucas, Dulcan, & Schwab-Stone, 2000) and 5 further questions from sections 17-19 of the Schedules for Clinical Assessment in Neuropsychiatry version 2.0 (World Health Organisation, 1994). The sum of all PLIKS items (definite and probable) was calculated along with subdomains scores for hallucinations, delusions and unusual perceptual experiences.

Four hundred and eighty-eight individuals completed both the SPQ and the PLIKSi. Many of the SPQ items had significant associations with psychotic experiences (total symptoms, hallucinations, delusions, and unusual perceptual experiences subscales). There were eight items that had at least a moderate effect size (Cramer’s V above .20) and were included in the analysis. These items were SPQ 4, 9, 13, 40, 55, 60, 61, and 63 (see Table C for full details of these items). We also individually considered an addition 10 SPQ items with a highly significant relationship and an effect size approaching the moderate level (Cramer’s V between .15 and .20). Of these ten, only three were included in the current analysis. The three items kept were SPQ28 (Have you ever noticed a common event or object that seemed to be a special sign for you?), SPQ31 (I often hear a voice speaking my thoughts aloud), and SPQ64 (Are your thoughts sometimes so loud that you can almost hear them?). These items were thought to have face validity as psychotic experiences, had a low prevalence of endorsement in those who did not endorse psychotic items at interview, were approximately twice as likely to be endorsed by those with psychotic experiences when compared with those without psychotic experiences and were chosen based on consensus between authors MCSC, IMG and PBJ.

# Details of Discriminant Validity Coding

Alcohol use was measured with three categories – never, occasional (any alcohol up to two days/week) and an often (3-5 days/week) combined with every day/nearly every day category; the most frequent category was rarely endorsed (1.53%). Cannabis and illegal drug (a class A, B or C drug, excluding cannabis) use were binary variables (no use/some use). Non-Suicidal Self-harm (NSSI) was coded in two ways – never versus more than once and never/once versus more than twice. The results were very similar for both approaches to coding but we report the results for the second coding, which distinguishes those who had multiple occurrences of past month NSSI.

Current and past treatment for emotional, behavioural or mental health difficulty was reported by either the participants (for those 18 or over) or the parent/guardian (for those under 18). Current and past mental health treatment were combined and two variables were created: experience of any type of mental health treatment and experience of treatment for depression. Treatment for primary physical disorders (e.g., epilepsy, heart palpitations, Crohn’s disease) were excluded.

# Fit indices by gender and age category

Table B. Latent trait modelling fit indices by gender and age category.

| Model | Chi Square | df | # parameters | CFI | TLI | RMSEA | WRMR |
| --- | --- | --- | --- | --- | --- | --- | --- |
| Girls |  |  |  |  |  |  |  |
| One factor model | 17739 | 5545 | 425 | .908 | .906 | .043 | 2.123 |
| 4 factor model | 11704 | 5834 | 469 | .957 | .956 | .029 | 1.530 |
| 5 factor model | 11337 | 5622 | 456 | .957 | .956 | .029 | 1.540 |
| SL 4 factor | 11037 | 5471 | 500 | .958 | .957 | .029 | 1.485 |
| ***SL 5 factor*** | ***10585*** | ***5350*** | ***510*** | ***.960*** | ***.959*** | ***.029*** | ***1.464*** |
| EBFA | 11476 | 5076 | 469 | .952 | .950 | .032 | 1.597 |
| Boys |  |  |  |  |  |  |  |
| One factor model | 13403 | 5651 | 427 | .925 | .923 | .037 | 1.857 |
| 4 factor model | 9811 | 5943 | 471 | .963 | .962 | .025 | 1.382 |
| 5 factor model | 9238 | 5729 | 458 | .966 | .965 | .024 | 1.352 |
| SL 4 factor | 9407 | 5471 | 500 | .962 | .960 | .027 | 1.379 |
| ***SL 5 factor*** | ***8432*** | ***5350*** | ***510*** | ***.970*** | ***.969*** | ***.024*** | ***1.273*** |
| EBFA | 9381 | 5177 | 472 | .959 | .957 | .028 | 1.442 |
| 14-15 |  |  |  |  |  |  |  |
| One factor model | 7115 | 5403 | 459 | .956 | .955 | .027 | 1.208 |
| 4 factor model | 7231 | 5834 | 468 | .965 | .964 | .024 | 1.129 |
| 5 factor model | 6870 | 5516 | 453 | .965 | .964 | .024 | 1.127 |
| SL 4 factor | 6691 | 5471 | 499 | .969 | .968 | .023 | 1.076 |
| ***SL 5 factor*** | ***6385*** | ***5247*** | ***506*** | ***.971*** | ***.969*** | ***.023*** | ***1.057*** |
| EBFA | 6497 | 5076 | 468 | .963 | .962 | .026 | 1.139 |
| 16-17 |  |  |  |  |  |  |  |
| One factor model | 7655 | 5403 | 460 | .953 | .952 | .030 | 1.256 |
| 4 factor model | 7467 | 5834 | 469 | .967 | .966 | .024 | 1.130 |
| 5 factor model* | 7008 | 5516 | 454 | .969 | .968 | .024 | 1.112 |
| SL 4 factor | 7071 | 5471 | 500 | .967 | .966 | .025 | 1.111 |
| ***SL 5 factor*** | ***6585*** | ***5247*** | ***507*** | ***.972*** | ***.971*** | ***.023*** | ***1.057*** |
| EBFA* | 6830 | 5076 | 469 | .963 | .962 | .027 | 1.156 |
| 18-19 |  |  |  |  |  |  |  |
| One factor model | 7593 | 5508 | 461 | .946 | .945 | .030 | 1.262 |
| 4 factor model | 7467 | 5834 | 468 | .959 | .958 | .025 | 1.150 |
| 5 factor model | 7170 | 5622 | 455 | .960 | .959 | .025 | 1.146 |
| SL 4 factor | 6995 | 5367 | 494 | .958 | .957 | .026 | 1.142 |
| ***SL 5 factor*** | ***6749*** | ***5350*** | ***509*** | ***.964*** | ***.963*** | ***.025*** | ***1.099*** |
| EBFA | 6849 | 5076 | 468 | .955 | .953 | .028 | 1.184 |
| 20-21 |  |  |  |  |  |  |  |
| One factor model* | 7491 | 5403 | 460 | .956 | .955 | .030 | 1.255 |
| 4 factor model | 7565 | 5834 | 469 | .964 | .963 | .026 | 1.175 |
| 5 factor model | 7144 | 5516 | 454 | .966 | .965 | .026 | 1.163 |
| SL 4 factor | 7084 | 5471 | 500 | .966 | .965 | .026 | 1.135 |
| ***SL 5 factor*** | ***6716*** | ***5247*** | ***507*** | ***.969*** | ***.968*** | ***.026*** | ***1.108*** |
| EBFA | 6754 | 5076 | 469 | .965 | .963 | .028 | 1.166 |
| 22-24 |  |  |  |  |  |  |  |
| One factor model | 7423 | 5403 | 460 | .965 | .964 | .028 | 1.244 |
| 4 factor model* | 7300 | 5834 | 469 | .975 | .974 | .023 | 1.121 |
| 5 factor model | 6965 | 5516 | 454 | .975 | .974 | .024 | 1.124 |
| SL 4 factor | 6914 | 5471 | 500 | .975 | .974 | .024 | 1.103 |
| ***SL 5 factor*** | ***6561*** | ***5247*** | ***507*** | ***.977*** | ***.976*** | ***.023*** | ***1.075*** |
| EBFA | 6524 | 4977 | 463 | .973 | .972 | .026 | 1.136 |

# Details of the final bifactor model: SL 5 specific bifactor model

Table C. Loadings for the general factor and five specific factors from the SL transformation of the 5 factor CFA. Severity thresholds^1^ for each item also given.

|  |  | General | Factor 1 | Factor 2 | Factor 3 | Factor 4 | Factor 5 | Positive Factor | Threshold 1 | | Threshold 2 | | Threshold 3 | | Threshold 4 |
| --- | --- | --- | --- | --- | --- | --- | --- | --- | --- | --- | --- | --- | --- | --- | --- |
| MFQ1 | Felt miserable or unhappy | 0.695 |  |  |  |  | 0.266 |  | -0.845 | 1.28 | | 2.385 | |  | |
| MFQ2 | Didn't enjoy anything | 0.632 |  |  |  |  | 0.246 |  | 0.21 | 1.663 | | 2.612 | |  | |
| MFQ5 | So tired/sat around and did nothing | 0.545 |  |  |  |  |  |  | -0.501 | 0.874 | | 1.933 | |  | |
| MFQ6 | Moving and walking more slowly than usual | 0.539 |  |  |  |  |  |  | 0.472 | 1.52 | | 2.33 | |  | |
| MFQ7 | Was very restless | 0.488 |  |  |  |  |  |  | -0.176 | 1.073 | | 1.926 | |  | |
| MFQ8 | Felt I was no good any more | 0.795 |  |  |  |  | 0.336 |  | 0.384 | 1.323 | | 1.913 | |  | |
| MFQ9 | Sometimes blamed myself for things not my fault | 0.709 |  |  |  |  |  |  | 0.141 | 1.265 | | 1.906 | |  | |
| MFQ11 | Got grumpy and cross easily | 0.608 |  |  |  |  |  |  | -0.613 | 0.683 | | 1.519 | |  | |
| MFQ12 | Felt like talking less than usual | 0.637 |  |  |  |  |  |  | -0.077 | 1.084 | | 1.84 | |  | |
| MFQ13 | Talking more slowly than usual | 0.556 |  |  |  |  |  |  | 0.996 | 1.892 | | 2.447 | |  | |
| MFQ14 | Cried a lot | 0.644 |  |  |  |  |  |  | 0.648 | 1.553 | | 2.199 | |  | |
| MFQ15 | Thought there was nothing good for me in future | 0.741 |  |  |  |  | 0.373 |  | 0.43 | 1.288 | | 1.879 | |  | |
| MFQ16 | Thought life was not worth living | 0.791 |  |  |  |  | 0.332 |  | 0.941 | 1.716 | | 2.187 | |  | |
| MFQ17 | Thought about dying | 0.701 |  |  |  |  |  |  | 0.757 | 1.767 | | 2.187 | |  | |
| MFQ18 | Thought my family would be better off without me | 0.741 |  |  |  |  | 0.225 |  | 1.103 | 1.828 | | 2.238 | |  | |
| MFQ19 | Thought about killing myself | 0.753 |  |  |  |  | 0.255 |  | 1.275 | 2.04 | | 2.426 | |  | |
| MFQ20 | Didn't want to see friends | 0.697 |  |  |  |  |  |  | 0.41 | 1.655 | | 2.298 | |  | |
| MFQ21 | Found it hard to think properly or concentrate | 0.735 |  |  |  |  |  |  | -0.516 | 0.908 | | 1.687 | |  | |
| MFQ22 | Thought bad things would happen to me | 0.768 |  |  |  |  |  |  | 0.385 | 1.564 | | 2.163 | |  | |
| MFQ23 | Hated myself | 0.827 |  |  |  |  | 0.333 |  | 0.57 | 1.397 | | 1.935 | |  | |
| MFQ24 | I was a bad person | 0.746 |  | 0.220 |  |  |  |  | 0.528 | 1.672 | | 2.225 | |  | |
| MFQ25 | Thought I looked ugly | 0.648 |  |  |  |  | 0.316 |  | -0.177 | 0.877 | | 1.583 | |  | |
| MFQ26 | Worried about aches and pains | 0.467 |  |  |  |  |  |  | -0.013 | 1.133 | | 1.805 | |  | |
| MFQ27 | Felt lonely | 0.714 |  |  |  |  | 0.247 |  | -0.116 | 0.969 | | 1.599 | |  | |
| MFQ28 | Thought nobody really loved me | 0.768 |  |  |  |  | 0.288 |  | 0.733 | 1.43 | | 1.997 | |  | |
| MFQ29 | Didn't have any fun at school/work | 0.625 |  |  |  |  |  |  | -0.008 | 1.077 | | 1.776 | |  | |
| MFQ30 | Thought I could never been as good as others | 0.772 |  |  |  |  | 0.239 |  | 0.118 | 1.004 | | 1.651 | |  | |
| MFQ31 | I did everything wrong | 0.836 |  |  |  |  |  |  | 0.505 | 1.516 | | 2.174 | |  | |
| MFQ32 | Didn’t sleep as well as usual | 0.532 |  |  |  |  |  |  | -0.308 | 0.744 | | 1.408 | |  | |
| RCMAS1 | Had trouble making up my mind | 0.596 |  |  |  |  |  |  | -0.531 | 0.84 | | 1.58 | |  | |
| RCMAS2 | Worried when things did not go the right way for me | 0.708 |  |  | 0.252 |  |  |  | -0.392 | 0.75 | | 1.527 | |  | |
| RCMAS3 | Others seem to do things more easily than me | 0.763 |  |  |  |  |  |  | -0.405 | 0.739 | | 1.452 | |  | |
| RCMAS4 | Trouble getting breath | 0.568 |  |  |  |  |  |  | 0.922 | 1.906 | | 2.612 | |  | |
| RCMAS5 | Worried a lot of the time | 0.781 |  |  | 0.494 |  |  |  | -0.187 | 0.703 | | 1.402 | |  | |
| RCMAS6 | Afraid of a lot of things | 0.780 |  |  | 0.365 |  |  |  | 0.348 | 1.209 | | 1.817 | |  | |
| RCMAS7 | Got angry easily | 0.641 |  |  |  |  |  |  | -0.103 | 0.894 | | 1.488 | |  | |
| RCMAS8 | Worried about what my parents would say | 0.631 |  |  |  |  |  |  | 0.333 | 1.211 | | 1.75 | |  | |
| RCMAS9 | Felt that others did not like the way I did things | 0.732 |  |  |  |  |  |  | 0 | 1.083 | | 1.756 | |  | |
| RCMAS10 | Hard for me to get to sleep at night | 0.555 |  |  |  |  |  |  | -0.199 | 0.738 | | 1.37 | |  | |
| RCMAS11 | Worried about what others thought about me | 0.755 |  |  |  |  |  |  | -0.426 | 0.638 | | 1.276 | |  | |
| RCMAS12 | Felt alone even when there were people with me | 0.813 |  |  |  |  |  |  | 0.287 | 1.089 | | 1.604 | |  | |
| RCMAS13 | Often felt sick to my stomach | 0.692 |  |  |  |  |  |  | 0.702 | 1.595 | | 2.04 | |  | |
| RCMAS16 | I was tired a lot | 0.622 |  |  |  |  |  |  | -0.753 | 0.396 | | 1.308 | |  | |
| RCMAS17 | Worried about what was going to happen | 0.780 |  |  | 0.347 |  |  |  | -0.273 | 0.822 | | 1.632 | |  | |
| RCMAS18 | Other people my age were happier than me | 0.801 |  |  |  |  | 0.213 |  | -0.267 | 0.724 | | 1.41 | |  | |
| RCMAS19 | Had bad dreams | 0.552 |  |  |  |  |  |  | 0.328 | 1.303 | | 1.899 | |  | |
| RCMAS20 | Feelings got hurt when I was fussed at | 0.756 |  |  |  |  |  |  | 0.364 | 1.186 | | 1.853 | |  | |
| RCMAS21 | Felt someone would tell me I did things the wrong way | 0.705 |  |  |  |  |  |  | 0.15 | 1.151 | | 1.898 | |  | |
| RCMAS22 | Wake up scared some of the time | 0.631 |  |  |  |  |  |  | 0.944 | 1.823 | | 2.314 | |  | |
| RCMAS23 | Worried when I went to bed at night | 0.673 |  |  | 0.309 |  |  |  | 0.248 | 1.08 | | 1.678 | |  | |
| RCMAS25 | Wiggled in my seat a lot | 0.475 |  |  |  |  |  |  | 0.012 | 0.889 | | 1.508 | |  | |
| RCMAS26 | Worried | 0.773 |  |  | 0.512 |  |  |  | -0.517 | 0.627 | | 1.26 | |  | |
| RCMAS27 | A lot of people were against me | 0.765 |  |  |  |  |  |  | 0.751 | 1.634 | | 2.187 | |  | |
| RCMAS28 | Worried about something bad happening to me | 0.744 |  |  | 0.239 |  |  |  | 0.336 | 1.296 | | 1.866 | |  | |
| LOI1 | Had to do things in a certain way to stop bad things happening | 0.535 |  |  |  | 0.532 |  |  | 0.891 | 1.475 | | 1.906 | |  | |
| LOI2 | Trouble finishing things as I had to do things over and over again | 0.580 |  |  |  |  |  |  | 0.697 | 1.455 | | 2.087 | |  | |
| LOI3 | Hated dirt and dirty things | 0.353 |  |  |  | 0.261 |  |  | 0.089 | 0.865 | | 1.396 | |  | |
| LOI4 | Special number to count to or did things a certain number of times | 0.382 |  |  |  | 0.620 |  |  | 1.168 | 1.628 | | 2.031 | |  | |
| LOI5 | Felt guilty about things even though no one thought I'd done anything wrong | 0.716 |  |  |  |  |  |  | 0.19 | 1.13 | | 1.649 | |  | |
| LOI6 | Worried about being clean enough | 0.488 |  |  |  | 0.280 |  |  | 0.349 | 1.195 | | 1.822 | |  | |
| LOI7 | Moved or talked in a special way to avoid bad luck | 0.376 |  |  |  | 0.666 |  |  | 1.063 | 1.62 | | 2.077 | |  | |
| LOI8 | Worried if I did something not exactly how I liked | 0.603 |  |  |  | 0.359 |  |  | 0.389 | 1.246 | | 1.816 | |  | |
| LOI9 | Fussy about keeping my hands clean | 0.341 |  |  |  | 0.286 |  |  | 0.456 | 1.163 | | 1.7 | |  | |
| LOI10 | had a special number or words that I said to keep bad luck away | 0.420 |  |  |  | 0.727 |  |  | 1.269 | 1.805 | | 2.128 | |  | |
| LOI11 | Thinking about things as wasn't sure they had been done the right way | 0.717 |  |  |  |  |  |  | -0.413 | 0.825 | | 1.53 | |  | |
| ABQ1 | Deliberately broke rules or disobeyed people | 0.422 |  | 0.660 |  |  |  |  | 0.494 |  | |  | |  | |
| ABQ2 | Stole things | 0.377 |  | 0.657 |  |  |  |  | 1.4 |  | |  | |  | |
| ABQ3 | Deliberately damaged property | 0.351 |  | 0.720 |  |  |  |  | 1.72 |  | |  | |  | |
| ABQ4 | Deliberately hurt or threatened someone | 0.400 |  | 0.637 |  |  |  |  | 1.488 |  | |  | |  | |
| ABQ5 | Skipped lessons/work or played truant for school/work | 0.343 |  | 0.470 |  |  |  |  | 0.711 |  | |  | |  | |
| ABQ6 | Deliberately lied or cheated to get what I want | 0.436 |  | 0.608 |  |  |  |  | 0.697 |  | |  | |  | |
| ABQ7 | Ran away from home | 0.504 |  | 0.504 |  |  |  |  | 1.893 |  | |  | |  | |
| ABQ11 | Deliberately hurt or been cruel to an animal | 0.307 |  | 0.516 |  |  |  |  | 2.14 |  | |  | |  | |
| RSE1 | At times, thought I was no good at all | 0.831 |  |  |  |  | 0.338 | 0.48 | 0.297 | 1.303 | | 1.859 | |  | |
| RSE2* | Was satisfied with myself | -0.607 |  |  |  |  | -0.365 |  | -1.227 | 0.01 | | 1.444 | |  | |
| RSE3* | Felt I had a number of good qualities | -0.554 |  |  |  |  | -0.377 |  | -1.426 | -0.116 | | 1.015 | |  | |
| RSE4* | Was able to do things as well as most people | -0.575 |  |  |  |  | -0.304 |  | -1.439 | -0.2 | | 1.002 | |  | |
| RSE5 | Felt I did not have much to be proud of | 0.723 |  |  |  |  | 0.395 | 0.587 | -0.081 | 1.055 | | 1.829 | |  | |
| RSE6 | Certainly felt useless at times | 0.802 |  |  |  |  | 0.325 | 0.540 | -0.107 | 1.068 | | 1.799 | |  | |
| RSE7* | Felt that I was as good as anyone else | -0.553 |  |  |  |  | -0.297 |  | -1.083 | 0.047 | | 0.936 | |  | |
| RSE8 | Wished I could have more respect for myself | 0.625 |  |  |  |  |  | 0.495 | -0.23 | 0.53 | | 1.177 | |  | |
| RSE9 | Felt I was a failure | 0.805 |  |  |  |  | 0.385 | 0.472 | 0.262 | 1.137 | | 1.811 | |  | |
| RSE10* | I took a positive attitude towards myself | -0.618 |  |  |  |  | -0.439 |  | -1.314 | -0.09 | | 0.85 | |  | |
| SPQ4 | Often mistaken objects for people or noises for voices? | 0.372 |  |  |  | 0.404 |  |  | 0.132 |  | |  | |  | |
| SPQ9 | I am sure I am being talked about behind my back | 0.603 |  |  |  |  |  |  | 0.266 |  | |  | |  | |
| SPQ13 | Had the sense of a person/force is around you, even if you can't see it? | 0.321 |  |  |  | 0.498 |  |  | 0.518 |  | |  | |  | |
| SPQ28 | Noticed a common event/object that seemed to be a special sign for you? | 0.338 |  |  |  | 0.472 |  |  | 0.837 |  | |  | |  | |
| SPQ31 | Often hear a voice speaking my thoughts aloud. | 0.336 |  |  |  | 0.407 |  |  | 1.002 |  | |  | |  | |
| SPQ40 | Seen things invisible to other people? | 0.359 |  |  |  | 0.535 |  |  | 1.405 |  | |  | |  | |
| SPQ60 | Sometimes feel that other people are watching you? | 0.531 |  |  |  | 0.349 |  |  | 0.394 |  | |  | |  | |
| SPQ61 | Suddenly feel distracted by distant sounds you're not normally aware of? | 0.406 |  |  |  | 0.496 |  |  | 0.492 |  | |  | |  | |
| SPQ63 | Sometimes feel that people are talking about you? | 0.537 |  |  |  | 0.212 |  |  | 0.167 |  | |  | |  | |
| SPQ64 | Thoughts sometimes so strong you can almost hear them? | 0.437 |  |  |  | 0.441 |  |  | 0.672 |  | |  | |  | |
| WB1* | Feeling optimistic about future | -0.474 | 0.313 |  |  |  | -0.465 | 0.195 | -1.878 | -0.95 | | -0.042 | | 1.152 | |
| WB2* | Feeling useful | -0.517 | 0.316 |  |  |  | -0.369 | 0.230 | -1.879 | -0.83 | | 0.169 | | 1.545 | |
| WB3* | Feeling relaxed | -0.573 | 0.424 |  |  |  |  | 0.186 | -1.828 | -0.783 | | 0.216 | | 1.481 | |
| WB5* | Had energy to spare | -0.395 | 0.397 |  |  |  |  | 0.217 | -1.479 | -0.399 | | 0.531 | | 1.561 | |
| WB6* | Been dealing with problems well | -0.582 | 0.415 |  |  |  |  | 0.350 | -1.823 | -0.927 | | 0.057 | | 1.359 | |
| WB7* | Been thinking clearly | -0.628 | 0.433 |  |  |  |  | 0.320 | -2.058 | -1.131 | | -0.121 | | 1.067 | |
| WB8* | Feeling good about myself | -0.656 | 0.383 |  |  |  | -0.404 | 0.288 | -1.817 | -0.91 | | 0.012 | | 1.138 | |
| WB9* | Feeling close to other people | -0.434 | 0.476 |  |  |  | -0.316 | 0.044 | -2.005 | -1.023 | | -0.137 | | 1.004 | |
| WB10* | Feeling confident | -0.570 | 0.421 |  |  |  | -0.369 | 0.293 | -1.762 | -0.87 | | 0.032 | | 1.167 | |
| WB11* | Been able to make up my own mind about things | -0.526 | 0.318 |  |  |  |  | 0.318 | -2.211 | -1.111 | | -0.209 | | 0.85 | |
| WB12* | Feeling loved | -0.503 | 0.406 |  |  |  | -0.342 | 0.032 | -1.872 | -1.12 | | -0.358 | | 0.516 | |
| WB13* | Been interested in new things | -0.368 | 0.424 |  |  |  | -0.242 | 0.163 | -1.972 | -1.17 | | -0.291 | | 0.803 | |
| WB14* | Been feeling cheerful | -0.633 | 0.480 |  |  |  | -0.318 | 0.160 | -2.014 | -1.157 | | -0.188 | | 1.011 | |

MFQ = Moods and Feelings Questionnaire; RCMAS = Revised Children’s Manifest Anxiety Scale; LOI = Leyton Obsessional Inventory; ABQ = Antisocial Behaviour Questionnaire; RSE = Rosenberg Self Esteem Scale; SPQ = Schizotypal Personality Questionnaire; WB = Warwick-Edinburgh Mental Well Being Scale. * = positively worded items.

^1^ The thresholds reported relate to the location of individual items along the latent general distress factor and the specific factors. Within a multivariate solution, as we have here, unidimensional thresholds are not possible and the threshold for each particular category could be reached by a high trait on the general distress, a specific factor, or any combination of the general or specific factors (Bock, Gibbons, & Muraki, 1988). Thus, the thresholds should be interpreted in terms of location across both the general as well as the relevant specific factors. The resulting thresholds indicate that for item 1 of the MFQ, for instance, the first threshold indicates where the probability of responding “sometimes” becomes larger than the probability of responding “never”. The second threshold indicates where the probability of responding “mostly/always” overtakes the probability of responding “sometimes”. All thresholds can be interpreted in a similar manner dependent on the original scale for each item.

# Further Details on the Analysis Procedure

The total sample (2,228 individuals) was randomly split into two sub-samples (by sorting all 2228 participant by random number, then creating two groups) for exploratory and confirmatory factor analysis, respectively. When fully confirmed, model fit was checked on the exploratory half (consistent with suggestions by Brown, 2006) and was found to be acceptable in all instances. The confirmatory procedure was then repeated on the full sample, producing slight alterations to create the final latent variable structure.

Two items on the ABQ (ABQ8: I threatened or forced someone to give their money or other belongings to me; ABQ9: I broke into someone else’s property (e.g. into a house or car)) were sparsely endorsed so were removed from all models because of difficulties with the covariance matrix. Additional ABQ items (usually ABQ11: I have deliberately hurt or been cruel to an animal (e.g., a pet)) were removed when needed from the subsample model fit checks reported in Table B as the smaller sample size caused additional covariance matrix difficulties.

As mentioned in the analysis section, the number of factors at the exploratory stage was chosen through evaluation of the eigenvalues and scree plot. Parallel analysis would have been preferable, but is unavailable within Mplus (Muthén & Muthén, 1998-2014) and was not computational possible in FACTOR (Lorenzo-Seva & Ferrando, 2006).

# Further details on the Exploratory Factor Analysis and Bi-Geomin ESEM

Within the exploratory phase, eigenvalues only dropped below one for 20 factors and dropped below two for eight factors. Four factors was the last solution to have an eigenvalue above 3, which also corresponded to a gradual break in the scree plot. However, as the scree plot was not definitive, we also considered a five and six factor solution, as well as the seven and 19 factor solution, consistent with an eigenvalue cut-off of 2 and 1, respectively. The four and five factor solutions had good construct validity, but the six and seven factor solutions had one factor containing high loadings only two related items (MFQ10: It was hard for me to make up my mind; RCMAS 1: I had trouble making up my mind). This was considered a spurious factor based on surface similarities between redundant items. There was no convergence for the 19 factor solution. Therefore, a one factor (for comparison purposes), four and five factor CFA solutions were fully evaluated.

For the ESEM Bi-Geomin solution, the scree plot indicated that either 4, 5 or 6 factors may be appropriate. The four factor solution contained a factor with high loadings on antisocial behaviour items as well as psychotic items, which as we mention in the main manuscript is not a widely reported finding in the literature and thus was considered theoretically implausible. The sixth factor solution again contained one factor containing only high loadings on the two items mentioned above. The five factor solution was considered theoretically plausible and was investigated further. Thus, a bifactor structure with one general factor and four specific factors was confirmed.

# Further details on the Confirmatory Factor Analysis

For the CFA structure, all loadings above .20 from the EFA were included on the factors, with the exception that any cross loading items below .25 were excluded unless both cross loading items were below .30. However, for some solutions, we additionally dropped the lower of the cross loadings when there were loadings on three factors or a large discrepancy between the highest and lowest loading (e.g., loadings of -.72 and -.40) in order to achieve convergence. We then consulted the modification indices to see if any relevant loadings were excluded from any factor. Any theoretically relevant loadings with a chi square change above 100 were added individually to the relevant factor. We required a high cut-off of 100 for chi square changes due to the large number of participants within this sample. We then evaluated the individual item characteristic curves on all loadings on the latent traits. We considered dropping items which had very poor discrimination on all relevant latent traits and also had very little of the item level variation accounted for by the factors, as measured by the communalities. For example, MFQ33 (I slept more than usual) was removed within the four factor CFA analysis due to low discriminability as well as the factors only accounting for 5.7% of the item level variance. Any item considered for deletion under this criteria but was considered theoretically important would have been retained. However, theoretically important items did not meet these criteria in any instance.

We then evaluated all non-significant loadings and removed all loadings which were non-significant. Throughout all of these procedures, ICC discrimination was evaluated with each loading removal. In the vast majority of cases, the item had very poor discrimination. We then evaluated items that had low loadings on a factor. We sequentially deleted items that were non-significant. We then evaluated and deleted items that had low loadings, deleting those with the lowest loadings first, then those closer to minimum loading of .25. We utilised a relatively low minimum loading in keeping with the general field of item level mental health factor analytic procedures (see Bohnke & Croudace, 2015; Brodbeck, Abbott, Goodyer, & Croudace, 2011; Chen, West, & Sousa, 2006). Indeed, in comparison to other studies, our approach was more stringent. In general, the field of mental health tends to use a lower cutoff than other fields, such as the measurement of IQ, as each indicator measuring a single aspect of mental health may carry a lot of weight for the majority of people, but no weight for a minority, leading the overall loading to be of middling value. The same could not be said of a measure of verbal intelligence, which should have a very strong relation to overall measure of intelligence or ‘g’ for everyone in the population.

After these procedures were concluded, we evaluated the occurrence of cross loading items. Any cross loadings below .30 (unless both were below .30) were removed individually. Cross loadings were also removed if there was a large discrepancy between the loadings (difference of .30), particularly if the lower cross loading did not theoretically fit with the other indicators on the factor.

The final step was to consult the modification indices once more for the presence of any correlated errors between individual indicators. A chi square change of 100 was the cut-off for considering whether to either delete a redundant item or included a correlated residual between two indicators. Consensus meetings between authors MCSC, IMG and PBJ decided whether the correlated items emerging for this analysis indicated redundant items, where one item should be deleted, or related, but distinct items, where a correlated residual should be included. For example, MFQ10 (it was hard for me to make up my mind) and RCMAS1 (I had trouble making up my mind) were considered redundant and worst fitting item (based on loading, communalities and ICC discrimination) was removed from the models (first order and bifactor).

The Confirmatory Bifactor Analysis (CBFA) form the ESEM exploratory bifactor proceeded on very similar grounds. However, as the general factor reduces loadings on the specific factors, a cutoff of .15 on the ESEM specific factors was used to create the confirmatory structure. Additionally, the final cutoff for the specific factors loading was .20.

Although we included a methods factor accounting for positive items in the analysis, we additionally added a second methods factor to account for the coding differences between the ABQ and the SPQ binary response categories and the remaining measures 4 or 5 response categories. There was an increase in model fit and no substantial deviations in the structure of the final model (5 factor SL Bifactor) when including this additional methods effect, which indicated the effect of different response formats was minimal.

# Details of items deleted from all fully confirmed models

Within each analysis, some items were deleted as per the analysis plan detailed above.

One factor first order model. MFQ3 (I was less hungry than usual), MFQ4 (I ate more than usual), MFQ10 (It was hard for me to make up my mind), MFQ33 (I slept more than usual), RCMAS14 (my feelings got hurt easily), RCMAS24 (Hard for me to keep my mind on work), LOI9 (Fussy about keeping my hands clean), and SPQ55 (Felt that you are communicating telepathically?) were removed during the CFA procedures.

Four factor first order model. For the four factor model, one item (MFQ4: I ate more than usual) was excluded at the EFA stage as it loaded below .20 on all factors. In total, 115 items were carried forward to the confirmatory factor analysis (CFA) stage. MFQ3 (I was less hungry than usual), MFQ33 (I slept more than usual), and ABQ11 (I have deliberately hurt or been cruel to an animal) were removed from the model during the CFA procedures.

Five factor first order model. MFQ3 (I was less hungry than usual) and RCMAS15 (My hands feel sweaty) were dropped from the analysis, as they did not load above 0.20 on the factors at the exploratory stage. MFQ4 (I ate more than usual), MFQ10 (Hard for me to make up my mind), MFQ33 (I slept more than usual), RCMAS14 (my feelings got hurt easily), and RCMAS24 (Hard for me to keep my mind on work) were removed from the model during the CFA procedures.

ESEM Exploratory and Confirmatory Bifactor analysis. MFQ4 (I ate more than usual), MFQ33 (I slept more than usual), and ABQ11 (I have deliberately hurt or been cruel to an animal (e.g. a pet)) were removed due to loadings below .30 on the general factor during the exploratory phase. The following items were deleted from the confirmation bifactor structure model due to low loadings (< .30) on the general factor: MFQ3 (I was less hungry than usual), SPQ55 (Have you ever felt that you are communicating with another person telepathically (by mind-reading)?), LOI9 (I was fussy about keeping my hands clean), SPQ13 (Have you ever had the sense that some person or force is around you, even though you cannot see anyone?), WB4 (feeling interested in other people) and SPQ31 (I often hear a voice speaking my thoughts aloud). Additionally, RCMAS15 (my hands feel sweaty), MFQ10 (it was hard for me to make up my mind), and RCMAS14 (my feelings got hurt easily) were removed following the confirming procedures.

Schmid-Leiman Bifactor Transformation of Four Factor CFA. For the SL bifactor transformation from the four factor CFA, we dropped any indicators which did not load at least .30 on the general factors (ABQ10: I have carried or used a weapon in a fight (e.g. a knife or a stick); SPQ55: Have you ever felt that you are communicating with another person telepathically (by mind-reading)?; WB04: I’ve been feeling interested in other people).

Schmid-Leiman Bifactor transformation of five factor CFA. For the SL bifactor transformation of the five factor CFA, three items were removed as they did not load above .30 on the general factor (ABQ10: I have carried or used a weapon in a fight (e.g. a knife or a stick); SPQ55: Have you ever felt that you are communicating with another person telepathically (by mind-reading)?; WB04: I’ve been feeling interested in other people).

# Latent Structural Analysis of the First Order Models: Further Details

One factor first order model. The loadings and factor structure for this model will be provided in a table format upon request, preferably to the first author. This model was not a good fit to the data, as can be seen in Table B. Therefore, we have ruled out the theoretical possible that a single factor may be the best fit to psychopathology symptoms.

Four factor first order model. Factor 1 from the four factor model consisted of 40 items and seems to represent broadly how individuals think of themselves. Positive wellbeing and self-esteem items load negative on this trait, and items measuring happiness, anhedonia, suicidality, negative self-comparison to others, feeling unloved, being lonely and hating themselves all loaded positively. Factor 2 consisted of 30 indicators and broadly seemed to represent psychopathological conditions excluding anxiety and depression. These items measured obsessional-compulsive behaviours, antisocial behaviours, and psychotic symptoms as well as items measuring sweaty hands and wiggling in their seat a lot, indicating some measurement of somatic symptoms. All items loaded positively. Factor 3 consisted of 28 items and broadly represented a worry dimension. These items measured thinking bad things would happen, generalised worry as well as specific worries (e.g., what parents would say, what other people thought of me, what was going to happen, about something bad happening to me, when things did not go the right way, when I go to bed). Additionally, this factor measured self-blame and guilt and rumination about previous actions. Factor 4 consisted of 15 items measuring somatic symptoms, including sleep and concentration problems, tiredness, talking less and more slowly than usual, not wanting to see friends, and getting angry easily. The loadings and factor structure for this model will be provided in a table format upon request, preferably to the first author.

Inter-factor correlations were all high. F2 was the least correlated with the other factors (F1: *r* = .67; F3: *r* = .77; F4: *r* = .70), but F1 and F3 (*r* = .89) and F3 and F4 (*r* = .86) were very highly correlated. F1 and F4 were also highly correlated (*r _­_*= .84). The very high correlations between F1 and F3 and F3 and F4 indicate that these factors may not be entirely independent (Brown, 2006) and the general pattern of very high correlations indicates a bifactor model may better account for the latent structure underlining these items.

Five factor first order model. The first factor was composed of 14 items and consisted of the well-being items, indicating this factor measures positive self-image. The second factor consisted of 11 indicators primarily measuring antisocial tendencies but also viewing yourself as a bad person and thinking people are against you. The third factor was composed of 37 items measuring somatic symptoms, concentration problems, generalised worry and more specific worries (e.g., worried when things don’t go right, about what parents will say, what others thought), concerns about how others view the participant, about others noticing the participant making mistakes, and negative self-comparison to others. The fourth factor consisted of 19 items broadly measuring aberrant thinking patterns, including obsessional/compulsive thoughts and psychotic symptoms. Factor five had 30 indicators broadly measuring negative mood, anhedonia, suicidality, negative self-evaluation (e.g., being a bad person, hating themselves, being no good at all, feeling useless and a failure), loneliness, and negative self-blame. There were negative loadings of positive self-esteem items on this factor. The loadings and factor structure for this model will be provided in a table format upon request, preferably to the first author.

The intercorrelations were all significant. F1 was correlated with F2 (*r* = -.33), F3 (*r* = -.75), F4 (*r* = -.51), and F5 (*r* = -.85). F2 was correlated with F3 (*r* = .56), F4 (*r* = .58), F5 (*r* = .49). F3 was correlated with F4 (*r* = .75) and F5 (*r* = .90). F4 was also correlated with F5 (*r* = .66). The factors that putatively measure more “internalising” aspects are the highest correlated and give cause for concern over whether these factors measure distinct latent traits (particularly F1-F5 and F3-F5).

Second order internalisation factor. To address the concerns above, we added a second order internalising factor to account for the correlations between F1, F3 and F5 detailed above. As can be seen in Table 1 and Table B this addition reduced the model fit and was not further evaluated.

# ESEM Exploratory and Confirmatory Bifactor analysis

The general factor consisted of 105 items and was considered this factor represents a latent dimension of distress. Positive items load negatively and negative items loading positively on this latent trait, indicating that higher scores are related to higher levels of distress. The loadings and factor structure, as well as IRT severity thresholds for each item as it loads on the general factor, for this model will be provided in a table format upon request, preferably to the first author.

Specific factor 1 contained nine items and generally measured worry and fear items as well as suicidal ideation items (life not worth living and thoughts about killing myself). Specific Factor 2 consisted of four items and generally measured restlessness, sleep difficulties and fatigue. Specific Factor 3 consisted of 18 items. This specific factor measured obsession-compulsive traits and psychotic experiences and could be thought of as a factor measuring aberrant thinking patterns. Specific factor 4 consisted of 14 items, which was dominated by positively loaded antisocial behaviour symptoms. Additionally, this factor consisted of a negative loading item about thinking they look ugly as well as positive self-esteem items.

# Schmid-Leiman Bifactor Transformation of Four Factor CFA.

The general factor loaded negative items positively and positive items negatively, such that a higher score in the general factor is equivalent to higher distress and lower positive self-image. A lower score related to lower distress and higher positive self-image. The loadings and factor structure, as well as IRT severity thresholds for each item as it loads on the general factor, for this model will be provided in a table format upon request, preferably to the first author.

Specific Factor 1 consisted of 18 items contained both positive and negative components. On the negative side (loading positively on the latent trait) were specific items related to negative comparison to others, feelings of worthlessness and feeling like a failure. The positive components were doing things as well as most people, having a positive attitude and being satisfied with self as well as being optimistic and confident about self and the future. Specific Factor 2 consisted of 27 items and was very similar to Factor 2 from the four factor CFA. This specific factor contained OCD items, antisocial behavioural items as well as psychotic symptoms. Specific Factor 3 contained 8 items about worry, fear and waking up scared. Specifically, worry about specific circumstances (thinking bad things would happen, worrying when things didn’t go the right way, and worrying when going to bed) loaded on this factor as well as more general worry questions (worried and worried a lot of the time). Additionally, bad dreams and waking up scared also loaded on this specific factor. Specific factor 4 consisted of 5 items related to sleep and fatigue problems.

# Age and Gender Differences: Full results

Table D. Full results of gender and age category differences.

| **Latent Trait** | Main Effects | Girls | Boys |
| --- | --- | --- | --- |
| **General Distress** |  |  |  |
| *Gender Main effect* | β= -.26, 95%CI(-.34,-.18), *p*<.001, η^2^= .02 |  |  |
| *Age main effect* | *F*(4,2222) = 3.444; *p* < .01 | *F*(4,1199) = 3.63; *p* < .01 | *F*(4,1019) = 3.56; *p* < .01 |
| *Gender by age interaction* | *F*(4,2218) = 3.74; *p* < .005 |  |  |
| *Specific age comparisons* |  |  |  |
| 14-15 to 16-17 |  | β= .20, 95%CI(.03,.37), *p*<.05, η^2^= .004 | β= .32, 95%CI(.13, .51), *p*<.005, η^2^= .01 |
| 16-17 to 18-19 |  | n.s. | β= -.22, 95%CI(-.40,-.04), *p*<.05, η^2^= .005 |
| 16-17 to 20-21 |  | β= .23, 95%CI(.06,.40), *p*<.01, η^2^= .006 | n.s |
| 14-15 to 20-21 |  | n.s. | β= .23, 95%CI(.05, .42), *p*<.05, η^2^= .006 |
| 14-15 to 20-21 |  | n.s. | β= .23, 95%CI(.05, .42), *p*<.05, η^2^= .006 |
| 16-17 to 22-24 |  | β= .26, 95%CI(.10,.42), *p*<.005, η^2^= .008 | n.s |
| 18-19 to 22-24 |  | β= .20, 95%CI(.03,.36), *p*<.05, η^2^= .005 | n.s |
| **Self-Confidence** |  |  |  |
| *Gender Main effect* | n.s. |  |  |
| *Age main effect* | *F*(4,2222) = 6.69; *p* < .001 | *F*(4,1199) = 4.42; *p* < .005 | *F*(4,1019) = 2.83; *p* < .05 |
| *Gender by age interaction* | N.s. |  |  |
| *Specific age comparisons* |  |  |  |
| 14-15 to 16-17 |  | β= -.15, 95%CI(-.31, -.003), *p*<.05, η^2^= .003 | n.s. |
| 14-15 to 20-21 |  | β= -.30, 95%CI(-.45,-.14), *p*<.001, η^2^= .01 | β= -.25, 95%CI(-.42,-.07), *p*<.01, η^2^= .008 |
| 14-15 to 22-24 |  | β = -.24, 95%CI(-.39,-.09), *p*<.005, η^2^= .008 | β= -.17, 95%CI(-.34,-.005), *p*<.05, η^2^= .004 |
| 16-17 to 20-21 |  | n.s. | β= .20, 95%CI(.03,.37), *p*<.05, η^2^= .005 |
| 18-19 to 20-21 |  | β= .18, 95%CI(.02,.33), *p*<.05, η^2^= .004 | β= .18, 95%CI(.01,.35), *p*<.05, η^2^= .004 |
| **Antisocial** |  |  |  |
| *Gender Main effect* | β= -.27, 95%CI(-.33,-.21), *p*<.001, η^2^= .04 |  |  |
| *Age main effect* | *F*(4,2222) = 10.22; *p* < .001 | *F*(4,1199) = 11.35; *p* < .001 | *F*(4,1019) = 3.40; *p* < .01 |
| *Gender by age interaction* | *F*(4,2218) = 2.63; *p* < .05 |  |  |
| *Specific age comparisons* |  |  |  |
| 14-15 to 20-21 |  | β= -.21, 95%CI(-.32,-.11), *p*<.001, η^2^= .01 | n.s |
| 14-15 to 22-24 |  | β= -.20, 95%CI(-.30,-.11), *p*<.001, η^2^= .01 | β= -.15, 95%CI(-.29,-.01), *p*<.05, η^2^= .005 |
| 16-17 to 18-19 |  | n.s | β= .16, 95%CI(.02,.30), *p*<.05, η^2^= .005 |
| 16-17 to 20-21 |  | β= .23, 95%CI(.12,.34), *p*<.001, η^2^= .01 | n.s |
| 16-17 to 22-24 |  | β= .22, 95%CI(.12,.33), *p*<.001, η^2^= .01 | β= .25, 95%CI(.11, .38), *p*<.001, η^2^= .01 |
| 18-19 to 20-21 |  | β= .24, 95%CI(.14,.32), *p*<.001, η^2^= .02 | n.s |
| 18-19 to 22-24 |  | β= .23, 95%CI(.13,.33), *p*<.001, η^2^= .02 | n.s |
| **Worry** |  |  |  |
| *Gender Main effect* | β= .24, 95%CI(.18,.31), *p*<.001, η^2^= .02 |  |  |
| *Age main effect* | *F*(4,2222) = 15.80; *p* < .001 | *F*(4,1199) = 10.59; *p* < .001 | *F*(4,1019) = 6.89; *p* < .001 |
| *Gender by age interaction* | n.s. |  |  |
| *Specific age comparisons* |  |  |  |
| 14-15 to 18-19 |  | n.s. | β= .24, 95%CI(.10,.39), *p*<.005, η^2^= .01 |
| 14-15 to 20-21 |  | β= .30, 95%CI(.16,.44), *p*<.001, η^2^= .01 | β= .29, 95%CI(.14,.44), *p*<.001, η^2^= .01 |
| 14-15 to 22-24 |  | β= .34, 95%CI(.20,.47), *p*<.001, η^2^= .02 | β= .26, 95%CI(.12,.40), *p*<.001, η^2^= .01 |
| 16-17 to 18-19 |  | n.s. | β= -.20, 95%CI(-.34,-.05), *p*<.001, η^2^= .007 |
| 16-17 to 20-21 |  | β= -.26, 95%CI(-.40,-.13), *p*<.001, η^2^= .01 | β= -.24, 95%CI(-.40,-.10), *p*<.005, η^2^= .01 |
| 16-17 to 22-24 |  | β= -.30, 95%CI(-.43,-.17), *p*<.001, η^2^= .02 | β= -.21, 95%CI(-.35,-.07), *p*<.005, η^2^= .008 |
| 18-19 to 20-21 |  | β= -.23, 95%CI(-.37,-.09), *p*<.005, η^2^= .009 | n.s. |
| 18-19 to 22-24 |  | β= -.27, 95%CI(-.40,-.13), *p*<.001, η^2^= .01 | n.s |
| **Aberrant Thoughts** |  |  |  |
| *Gender Main effect* | n.s. |  |  |
| *Age main effect* | *F*(4,2222) = 10.64; *p* < .001 | *F*(4,1199) = 6.16; *p* < .001 | *F*(4,1019) = 5.79; *p* < .001 |
| *Gender by age interaction* | n.s. |  |  |
| *Specific age comparisons* |  |  |  |
| 14-15 to 16-17 |  | (β= -.17, 95%CI(-.32,-.03), *p*<.05, η^2^= .005 | n.s. |
| 14-15 to 18-19 |  | β= -.21, 95%CI(-.35,-.06), *p*<.01, η^2^= .006 | n.s. |
| 14-15 to 20-21 |  | β= -.20, 95%CI(-.34,-.05), *p*<.01, η^2^= .006 | n.s. |
| 14-15 to 22-24 |  | β= -.36, 95%CI(-.50,-.21), *p*<.001, η^2^= .02 | β = -.28, 95%CI(-.42,-.13), *p*<.001, η^2^= .01 |
| 16-17 to 20-21 |  | n.s. | (β= .14, 95%CI(.003,.29), *p*<.05, η^2^= .004 |
| 16-17 to 22-24 |  | β= .18, 95%CI(.05, .32,), *p*<.01, η^2^= .006 | β= .30, 95%CI(.16, .44), *p*<.001, η^2^= .02 |
| 18-19 to 22-24 |  | β= .15, 95%CI(.10, .29), *p*<.05, η^2^= .004 | β= .26, 95%CI(.11, .40), *p*<.005, η^2^= .01 |
| 20-21 to 22-24 |  | β= .16, 95%CI(.02, .30), *p*<.05, η^2^= .004 | β= .16, 95%CI(.01, .30), *p*<.05, η^2^= .004 |
| **Mood** |  |  |  |
| *Gender Main effect* | n.s. |  |  |
| *Age main effect* | *F*(4,2221) = 2.68; *p* < .05 | *F*(4,1198) = 2.05; *p* = .09 | *F*(4,1018) = 3.08; *p* < .05 |
| *Gender by age interaction* | *F*(4,2217) = 2.59; *p* < .05 |  |  |
| *Specific age comparisons* |  |  |  |
| 14-15 to 16-17 |  | n.s | β=.17, 95%CI(.02, .32), *p*<.05, η^2^= .005 |
| 14-15 to 18-19 |  | n.s | β= .17, 95%CI(.01, .33), *p*<.05, η^2^= .004 |
| 14-15 to 22-24 |  | n.s | β= .27, 95%CI(.12, .42), *p*<.001, η^2^= .01 |

# Latent Profile Analysis: Further details

After exploration of the best latent construct to explain the psychopathology and mental wellbeing symptoms, we considered the possibility that there may be distinctive sub-populations that can be classified by examining patterns across the general and specific factors. This is achieved using latent profile analysis, which we have previously applied to self-reported depressive symptoms together with morning cortisol levels (Owens et al., 2014).

The fit statistics for 2 to 7 classes are shown in Table 2 of the main manuscript and Table E for the two age split analyses. Six subgroups were considered the best fit for the overall analysis as the AIC, BIC and BIS-SSA were lowest for the 6 class model. Additionally, the seven class solution did not show improved model fit according to the VLMR and LMR adjusted tests, while the comparable test for the six class solution showed a significant improvement over the five class solution.

Table E. Fit statistics of the LPA solutions for only those 18 and under and only those 19 and over.

| **18 and Under** | **Log Likelihood** | **AIC** | **BIC** | **BICSSA** | **Entropy** | **VLMR test** | **LMR Adj** | **Bootstrap** |
| --- | --- | --- | --- | --- | --- | --- | --- | --- |
| Two classes | -8355.21 | 16748.42 | 16844.74 | 16784.39 | 0.717 | -- | -- | <.001 |
| Three classes | -8322.47 | 16696.93 | 16828.75 | 16746.16 | 0.673 | 0.111 | 0.115 | <.001 |
| Four classes | -8252.74 | 16571.49 | 16738.79 | 16633.97 | 0.761 | 0.240 | 0.246 | <.001 |
| Five classes | -8186.70 | 16453.41 | 16656.20 | 16529.15 | 0.807 | 0.122 | 0.125 | <.001 |
| Six classes | -8209.95 | 16513.90 | 16752.18 | 16602.89 | 0.784 | 0.262 | 0.270 | <.001 |
| **19 and Over** |  |  |  |  |  |  |  |  |
| Two classes | -7310.32 | 14658.64 | 14752.85 | 14692.51 | 0.841 | -- | -- | -- |
| Three classes | -7270.59 | 14593.19 | 14722.11 | 14639.53 | 0.712 | 0.052 | 0.055 | <.001 |
| Four classes | -7190.27 | 14446.53 | 14610.16 | 14505.35 | 0.788 | 0.010 | 0.011 | <.001 |
| Five classes | -7142.41 | 14364.82 | 14563.16 | 14436.11 | 0.834 | 0.041 | 0.044 | <.001 |
| Six classes | -7112.54 | 14319.08 | 14552.13 | 14402.85 | 0.796 | 0.019 | 0.021 | <.001 |
| Seven classes | -7098.87 | 14305.74 | 14573.50 | 14401.98 | 0.751 | 0.550 | 0.557 | <.001 |

See Table F for the gender distribution and average age within each of the six subgroups.

Table F. Gender distribution and average age for all six subgroups.

|  | % (n) Female | Age (SD) |
| --- | --- | --- |
| Subgroup 1 | 35.8% (58/162) | 18.35 (2.84) |
| Subgroup 2 | 77.2% (267/346) | 19.61 (2.93) |
| Subgroup 3 | 52.3% (466/891) | 19.38 (3.16) |
| Subgroup 4 | 61.8% (259/419) | 19.06 (2.90) |
| Subgroup 5 | 39.3% (146/372) | 18.39 (2.73) |
| Subgroup 6 | 21.1% (8/38) | 19.58 (2.53) |
| Total | 54.0% (1104/2228) | 19.11 (3.01) |

# Latent profile analysis: Five class solution for those 18 and under

As the age category findings indicated there were developmental differences in trait levels, we chose to evaluate two further latent profile analyses to determine whether there were developmental shifts in the subgroups dependent on age. We split the sample at the 18/19 split giving us a relatively equal sample of approximately 1000 individuals for each analysis.

The best fitting model for those 18 and under indicated six subgroups (see Table E). Subgroup 1 had high levels of antisocial traits and reduced levels of worry. Subgroup 2 had high levels of distress associated with low levels of antisocial traits and high levels of worry. Subgroup 3 had reduced levels of general distress and antisocial behaviour. Subgroup 4 had high levels of general distress only. Subgroup 5 had high levels high antisocial traits, low worry and moderately high levels of aberrant thinking. Subgroup 5 had very high levels of antisocial behaviour, high self-confidence and aberrant thinking and low worry traits. Overall, there was a striking correspondence to the full sample LPA analysis. See Fig A.

Fig A. Latent Trait Levels for the six subgroup latent profile analysis for participants aged 18 and under.

# Latent profile analysis: Six class solution for those 19 and over

The best fitting model for those aged 19 and over indicated six subgroups (see Table E). Subgroup 1 had high levels of antisocial traits only. Subgroup 2 had very high levels of distress, lower self-confidence, antisocial traits and aberrant thoughts, and high worry. Subgroups 3 had lower general distress and antisocial traits. Subgroup 4 had high distress and antisocial traits. Subgroup 5 had high distress, self-confidence and aberrant thoughts, and reduced levels of antisocial traits and levels of mood. Subgroup 6 was defined by high levels general distress, self-confidence, antisocial behaviour and aberrant thoughts, with reduced levels of worry. In generally, the patterns again correspond well to the overall LPA solution, with the exception of subgroups five, which showed elevated levels of antisocial behaviour in the younger and full group LPA solutions, but reduced antisocial tendencies in those over 18. See Fig B.

Fig B. Latent Trait Levels for the six subgroup latent profile analysis for participants aged 19 and over.

# References

Bock, R. D., Gibbons, R., & Muraki, E. (1988). Full-Information Item Factor-Analysis. *Applied Psychological Measurement, 12*(3), 261-280. doi: Doi 10.1177/014662168801200305

Bohnke, J. R., & Croudace, T. J. (2015). Calibrating well-being, quality of life and common mental disorder items: psychometric epidemiology in public mental health research. *Br J Psychiatry*. doi: 10.1192/bjp.bp.115.165530

Brodbeck, J., Abbott, R. A., Goodyer, I. M., & Croudace, T. J. (2011). General and specific components of depression and anxiety in an adolescent population. *BMC Psychiatry, 11*, 191. doi: 10.1186/1471-244X-11-191

Brown, T. A. (2006). *Confirmatory Factor Analysis for Applied Research*. New York, NY: The Guilford Press.

Chen, F. F., West, S. G., & Sousa, K. H. (2006). A comparison of bifactor and second-order models of quality of life. *Multivariate Behavioral Research, 41*(2), 189-225. doi: DOI 10.1207/s15327906mbr4102_5

Horwood, J., Salvi, G., Thomas, K., Duffy, L., Gunnell, D., Hollis, C., . . . Harrison, G. (2008). IQ and non-clinical psychotic symptoms in 12-year-olds: results from the ALSPAC birth cohort. *Br J Psychiatry, 193*, 185-191. doi: 10.1192/bjp.bp.108.051904

Lorenzo-Seva, U., & Ferrando, P. J. (2006). FACTOR: A computer program to fit the exploratory factor analysis model. *Behavior Research Methods, 38*(1), 88-91. doi: Doi 10.3758/Bf03192753

Muthén, B., & Muthén, L. (1998-2014). *Mplus User's Guide. Seventh Edition*. Los Angeles, CA: Muthén & Muthén.

Owens, M., Herbert, J., Jones, P. B., Sahakian, B. J., Wilkinson, P. O., Dunn, V. J., . . . Goodyer, I. M. (2014). Elevated morning cortisol is a stratified population-level biomarker for major depression in boys only with high depressive symptoms. *Proc Natl Acad Sci U S A, 111*(9), 3638-3643. doi: 10.1073/pnas.1318786111

Shaffer, D., Fisher, P., Lucas, C. P., Dulcan, M. K., & Schwab-Stone, M. E. (2000). NIMH Diagnostic Interview Schedule for Children Version IV (NIMH DISC-IV): description, differences from previous versions, and reliability of some common diagnoses. *J Am Acad Child Adolesc Psychiatry, 39*, 28-38. doi: 10.1097/00004583-200001000-00014

World Health Organisation. (1994). *Schedules for Clinical Assessment in Neurospychiatry*: American Psychiatric Research.
